# Comprehensive multi-omics analysis and experimental validation indicate that VPS35 is a promising biomarker for prognosis, immunotherapy, and chemotherapy in LIHC

**DOI:** 10.1039/d5md00834d

**Published:** 2026-01-12

**Authors:** Dan Wan, Yajie Qi, Yali Kang, Fanfan Liang, Qian Wang, Hailong Zhang, Shaoying Zhang, Xiao Liang

**Affiliations:** a National and Local Joint Engineering Research Cente of Biodiagnosis and Biotherapy, The Second Affiliated Hospital of Xi'an Jiaotong University Xi'an 710004 China shaw0923@xjtu.edu.cn; b Shaanxi Provincial Clinical Medical Research Center for Liver and Spleen Diseases, The Second Afffliated Hospital of Xi'an Jiaotong University Xi'an 710004 China; c Tumor and Immunology Center of Precision Medicine Institute, Xi'an Jiaotong University Xi'an 710004 China

## Abstract

***Background***: Vacuolar protein sorting 35 (*VPS35*) is a key subunit of the retromer complex, and several previous studies have shown that *VPS35* plays an important role in the progression of hepatocellular carcinoma (LIHC). However, the comprehensive value of *VPS35* in LIHC in prognostic assessment, immunotherapy and chemotherapy has not been systematically reported. ***Methods***: To characterize the comprehensive value of *VPS35* in LIHC, we performed a comprehensive multi-omics analysis. Based on multiple databases such as The Cancer Genome Atlas (TCGA), Gene Expression Omnibus (GEO), and Human Protein Atlas (HPA), the relevant analyses were accomplished using R software and websites such as TIMER2, STRING, and TISIDB. We primarily evaluated the correlation of VPS35 expression in LIHC with prognosis, immune microenvironment infiltration, immunotherapy and chemotherapy. Western blotting (WB), quantitative real-time PCR (RT-qPCR), CCK8, colony-formation assay, Transwell, and *in vivo* experiments were also performed to verify the function of VPS35 in LIHC. ***Results***: Compared with normal tissues, *VPS35* was highly expressed in a variety of tumor tissues such as LIHC. *VPS35* has a good diagnostic value in a variety of tumors. In a variety of tumors such as LIHC, upregulation of *VPS35* expression levels is associated with poor prognosis. In LIHC, *VPS35* expression levels were significantly correlated with clinicopathological features such as the T stage, pathological stage, histological grade, vascular invasion, and residual tumor. Pan-cancer analysis showed that *VPS35* expression was positively correlated with tumor mutation burden (TMB) in 14 cancer types and microsatellite instability (MSI) in 15 cancer types. GO, KEGG and gene set enrichment analysis (GSEA) revealed that *VPS35* was positively correlated with the immunoglobulin complex, pro-iso-cellular adhesion *via* plasma membrane adhesion molecules, and immunoglobulin receptor binding. Multiple related genes of *VPS35* also had strong prognostic value in LIHC. Immune infiltration analysis showed that *VPS35* expression levels were associated with multiple immune cell infiltrations. Further analysis showed that *VPS35* expression levels were higher in proliferating T cells (Tproif) and monocyte-derived macrophages in LIHC. Downregulation of *VPS35* expression levels may enhance the therapeutic efficacy of immune checkpoint inhibitors (ICIs). Knockdown of *VPS35* significantly reduced the proliferation, invasion, migration of tumor cells and inhibited subcutaneous tumor formation. ***Conclusion***: *VPS35* is highly expressed in LIHC and exhibits significant diagnostic and prognostic value. Targeted knockdown of *VPS35* may inhibit LIHC progression and enhance the efficacy of immunotherapy and chemotherapy.

## Abstract 2

Vacuolar protein sorting 35 (*VPS35*) is highly expressed in LIHC and exhibits significant diagnostic and prognostic value. Targeted knockdown of *VPS35* may inhibit LIHC progression and enhance the efficacy of immunotherapy and chemotherapy.

## Graphical abstract

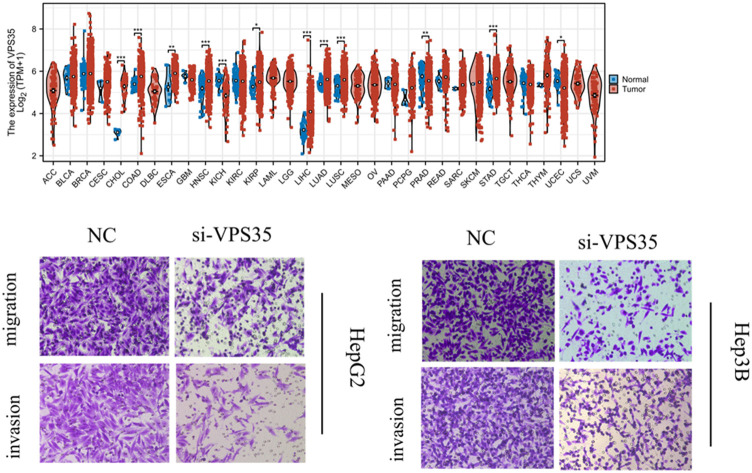

## Introduction

LIHC accounts for more than 80% of primary liver cancers, which is the sixth most common cancer and the third leading cause of cancer-related death globally.^1^ Asia, Europe, Africa, North America, and South America contribute >70%, 9.7%, 6.01%, 5.1%, and 4.4% of global LIHC incidence, respectively.^1^ It was estimated that 1.4 million individuals could be diagnosed and 1.3 million individuals could die from liver cancer globally in 2040.^2^ Most patients with LIHC are diagnosed at advanced stages.^1^ Despite significant advancements that have been made in the treatment of LIHC, such as liver transplantation, hepatic resection, immunotherapy, and targeted therapy,^3^ the 5-year recurrence rate of LIHC after hepatic resection was up to nearly 70%.^4^ Therefore, identifying diagnostic and therapeutic strategies for liver cancer has become an urgent public health priority. The development and progression of LIHC are accompanied by the abnormal expression of numerous genes. Previous studies have shown that many genes play critical roles in LIHC progression, chemotherapy resistance, immune evasion, and other behaviors,^5–8^ however, the specific mechanisms underlying various malignant biological behaviors of LIHC remain incompletely understood. Therefore, further exploration of novel biomarkers capable of accurately predicting LIHC prognosis, chemotherapy resistance, immune evasion, and other malignant behaviors is of great significance for the prevention and treatment of LIHC.

*VPS35* is a key subunit of the retromer complex, which plays a vital role in endosomal protein sorting. The retromer complex, a highly conserved complex, is primarily responsible for retrograde transport, mediating the retrieval of specific cargo proteins (*e.g.*, receptors, transporters) from endosomes back to the *trans*-Golgi network or to the plasma membrane.^9,10^ This process is crucial for maintaining cellular homeostasis, including receptor recycling, nutrient sensing, and developmental signaling.^10^ As the core scaffolding subunit of the retromer, *VPS35* is essential for recognizing and binding to a diverse set of cargo proteins, thereby dictating the specificity of retrograde sorting.^9,11^ It has been demonstrated that *VPS35* was implicated in neurodegenerative diseases and tumor progression.^12,13^ A recent study identified that *VPS35* was a novel oncogene, and it was upregulated in breast cancer, gastric cancer, and LIHC.^14,15^*VPS35* affects the development and metastasis of cancers by regulating a variety of factors and pathways. For instance, *VPS35* is critical in breast cancer progression and influences the autophagy process.^13^ Besides, *VPS35* promotes gastric cancer cell proliferation by recycling EGFR to the cell surface, leading to the downstream activation of the ERK1/2 pathway. In addition, *VPS35* was involved in tumor growth, invasion, and metastasis^16,17^ of LIHC. Considering the unfavorable prognosis and treatment resistance of LIHC, it is necessary to explore whether *VPS35* could serve as a prognostic biomarker and therapeutic target for immunotherapy and chemotherapy in LIHC.

The present study aims to explore the role of *VPS35* in the development of LIHC. We first evaluated the expression and clinicopathological characteristics and prognosis of *VPS35* in LIHC based on the TCGA and GEO database, followed by necessary experiments in clinical LIHC samples and LIHC cell lines to validate the characterization and function of *VPS35* in LIHC. Our study results provided novel clues for understanding the mechanism of *VPS35* affecting LIHC progression and prognosis.

## Materials and methods

### Data acquisition and processing

Gene expression arrays and clinical information of LIHC were driven from the TCGA database (https://portal.gdc.cancer.gov/), which consisted of 374 LIHC patients and 50 normal liver tissue cases. Patient selection criteria: the TCGA-LIHC cohort was pre-screened by TCGA based on histopathological confirmation of primary hepatocellular carcinoma. From the initial dataset, we applied the following criteria: inclusion: availability of *VPS35* expression data (RNA-Seq FPKM values), complete records for at least one clinicopathological variable (*e.g.*, TNM stage, grade, survival status). Exclusion: outlier expression values (defined as ±3 SD from the mean), cases with missing critical clinical data (*e.g.*, undefined stage, unknown vital status). RNAseq data in HTSeq-FPKM format was converted to TPM format and then log 2(*x* + 1) transformations were conducted.

Besides, three GEO datasets (GSE76427, GSE84598, and GSE45267) were obtained from GEO database (https://www.ncbi.nlm.nih.gov/geo/), which were subsequently analyzed and visualized using the ggplot2 package in R. Immunohistochemical images of LIHC and normal liver tissues were downloaded from the Human Protein Atlas (HPA) database (https://www.proteinatlas.org/). We analyzed the expression of *VPS35* using SpatialTME (https://www.spatialtme.yelab.site/# four LIHC samples' spatial transcriptome data were obtained in (!/))^18^ to analyze the expression of *VPS35*. They are HCC_Pmid34919432-HCC-1L, HCC_Pmid34919432-HCC-2L, HCC_Pmid34919432-HCC-3L, and HCC_Pmid34919432-HCC-4L, respectively.

### Patients and tissue samples

Five cases of LIHC tumor tissues and corresponding adjacent nontumor tissues (located >3 cm away from the tumor edge) were collected from patients undergoing surgical resection at the Second Affiliated Hospital of Xi'an Jiaotong University. The inclusion criteria were: (1) histopathologically confirmed primary hepatocellular carcinoma; (2) no preoperative radiotherapy or chemotherapy; (3) availability of complete clinical and pathological data. The basic clinicopathological characteristics of these patients are summarized in Table S1, including age, gender, TNM stage, and histologic grade. This cohort comprised patients with varying disease stages, enhancing the representativeness of our samples. This study was approved by the Ethics Committee of the Second Affiliated Hospital of Xi'an Jiaotong University. Human tissue was used in strict accordance with the guidelines of the Declaration of Helsinki, and the patients provided written informed consent to participate in this study.

### Cell line culture

In our research, human liver cancer cells HepG-2 and Hep3B were obtained from the Shanghai Institute for Biological Sciences' Cell Resource Center. The cells were subsequently cultured in DMEM medium (manufactured by Gibco BRL, USA), containing 10% fetal bovine serum (FBS) sourced from Corning, and a 1% penicillin–streptomycin solution provided by Biosharp. The cells were cultured in a growth environment of 37 °C and 5% CO_2_ in a CO_2_ incubator.

### The diagnostic and prognostic value of VPS35 in pan-cancer

The receiver operating characteristic (ROC) curve was employed to assess the potential diagnostic value of *VPS35* in pan-cancer. The visualization was completed using the pROC and ggplot2 packages in R. Moreover, univariate Cox regression analysis was used to assess the correlation of VPS35 expression with overall survival (OS), disease-specific survival (DSS), and progression-free interval (PFI) in pan-cancer by the survival and ggplot2 package in R. The patients with LIHC were classified into high- and low-expression groups based on the median expression level of *VPS35*. Kaplan–Meier curve analysis for OS, DSS, and PFI in LIHC patients stratified by *VPS35* expression was conducted using the survival packages in R. Risk factor analyses were conducted using the “ggrisk” packages, and the results were visualized using the “ggplot2” package.

### The correlation between VPS35 expression and clinicopathological features in LIHC

The correlation between VPS35 expression and clinical pathological characteristics, including age, gender, BMI, AFP, bilirubin, tumor staging, grading, and vascular invasion, was analyzed in LIHC tissues. The visualization was completed using the “ggplot2” package.

### The mutational analysis of VPS35 in pan-cancer

Based on the cBioPortal database (https://www.cbioportal.org),^19^ we analyzed the genetic alteration frequencies of *VPS35* across pan-cancer. Using TCGA data, we analyzed the correlations of VPS35 expression levels with tumor mutational burden (TMB) and microsatellite instability (MSI) in various cancer types. For LIHC, we obtained STAR-counts data, mutation MAF files, and corresponding clinical information from TCGA. The TPM-formatted data were extracted from STAR counts and normalized using log 2 (TPM + 1) transformation. In R software, we utilized the map tools package to download and visualize somatic mutation data from LIHC patients. Additionally, we investigated copy number variations (CNV) of *VPS35* in LIHC through the GSCA database (https://guolab.wchscu.cn/GSCA/#).^20^

### The differential expression gene and enrichment analysis of VPS35 in LIHC

LIHC patients were stratified into high- and low-*VPS35* expression groups based on median expression levels. Differential expression analysis between the two groups was conducted using the LIMMA package in R, employing empirical Bayes linear models to analyze the expression matrix. Differentially expressed genes (DEGs) were defined as those meeting the threshold criteria of |log 2 (fold change)| > 1.5 with an adjusted false discovery rate (FDR) < 0.05. Functional enrichment analyses, including gene ontology (GO), KEGG, and gene set enrichment analysis (GSEA), were subsequently performed using the clusterProfiler package to elucidate the biological significance of the identified DEGs between two groups. The C2.CP.KEGG gene set (v7.2, curated subset from MSigDB) was utilized as the reference gene set database. A significantly enriched pathway was identified using a combined threshold of normalized enrichment score (NES), false discovery rate (FDR) < 0.25, and adjusted *P* < 0.05.

### The related genes and PPI network analysis of VPS35 in pan-cancer

We first obtained the protein–protein interaction (PPI) network of *VPS35* from the STRING database and visualized the top 30 genes with the highest interaction scores. In addition, we used R software to analyze the co-expressed genes of *VPS35* in LIHC and visualized the top 30 co-expressed genes. Furthermore, we extracted the top 10 genes with the highest interaction scores and the top 10 genes with the highest correlation coefficients and analyzed their co-expression patterns in the STRING database (https://string-db.org/).^21^ Finally, we performed correlation analysis on these genes in R and visualized the results using the “circle” package.

### The prognostic value of VPS35-related genes in LIHC

First, we analyzed the prognostic value of the aforementioned 20 genes in LIHC. In addition, we selected the genes with prognostic significance in LIHC and examined their expression levels across different tumor stages using the GSCA database (https://guolab.wchscu.cn/GSCA).

### The correlation between VPS35 expression and the tumor immune microenvironment (TIME) in pan-cancer

Using TISIDB (http://cis.hku.hk/TISIDB/index.php),^22^ we explored how *VPS35* modulated tumor–immune interactions by assessing its association with tumor-infiltrating lymphocytes (TILs), immune stimulators, immune inhibitors, the major histocompatibility complex (MHC) molecule, chemokines, and receptors.

### The single-cell expression analysis of VPS35 in LIHC

To investigate the expression of *VPS35* in immune cells within the tumor immune microenvironment, we analyzed its expression profile using the Tumor Immune Single-Cell Hub 2 (TISCH2) database (http://tisch.comp-genomics.org/).^23^

### The immunotherapy and chemotherapy response analysis of VPS35 in LIHC

RNA-sequencing expression profiles and relevant clinical data for LIHC were obtained from the TCGA dataset. Subsequently, the tumor immune dysfunction and exclusion (TIDE) algorithm was utilized to predict potential responses to immune checkpoint inhibitor (ICI) therapy. In addition, the chemotherapy response in LIHC patients with high *versus* low *VPS35* expression was predicted using the Genomics of Drug Sensitivity in Cancer (GDSC) and Cancer Therapeutics Response Portal (CTRP) databases. Drug sensitivity prediction was performed using the prophetic R package, with half-maximal inhibitory concentration (IC_50_) values estimated through ridge regression analysis.

### The correlation of VPS35 with ferroptosis and m6A-related genes in LIHC

Ferroptosis refers to the accumulation of iron and lipid hydroperoxides, which could induce cell death. m6A is a type of RNA methylation, which specifically refers to the methylation modification at the 6th nitrogen atom of adenosine (A). The data of ferroptosis-related genes and m6A-associated genes were driven from the published research by Liu *et al.*^24^ and Li *et al.*,^25^ respectively. The patients with LIHC were categorized into high- and low-expression groups according to the median expression level of *VPS35*. The expression levels of ferroptosis and m6A-related genes were compared between the two groups.

### Molecular docking based on VPS35

We located the *VPS35* gene on the Coremine medical ontology information retrieval platform (https://coremine.com/medical/)^26^ and screened for potential regulatory molecules. From the PubChem database (https://pubchem.ncbi.nlm.nih.gov/)^27^ we obtain the molecular structure of the compound.

### RNA extraction and quantitative real-time PCR (RT-qPCR)

Firstly, both tissue samples and transfected cells were homogenized in TRIzol™ reagent (Invitrogen) for RNA isolation by the manufacturer's instructions. Subsequently, cDNA synthesis was carried out using a PrimeScript RT reagent kit (Takara). The mRNA expression levels were determined using the Takara SYBR Premix Ex Taq II kit, normalized to GAPDH and calculated using the 2^−ΔΔCt^ method. The experiments were performed with three independent biological replicates (*n* = 3). The nucleotide sequences used in this study are listed below:

GAPDH-forward: TGTGGGCATCAATGGATT TGG

GAPDH-reverse: ACACCATGTATTCCGGGTC AAT

VPS35-forward: GTTTTGACTGGCATATTGGAGCA

VPS35-reverse: TCTGGTGTAACTCAGCACAGG

### Western blotting

Firstly, the tissue or cells were dissolved in RIPA lysis buffer (Beyotime Biotechnology, China) and incubated in a 4 °C refrigerator for 20 minutes, gently pipetting the tissue lysis buffer to facilitate the complete lysis of the tissue. After the samples were centrifuged for 20 min at 13 000*g*, the protein concentration of the supernatant was determined using the BCA assay kit (Beyotime, Jiangsu, China). 12% sodium dodecyl sulfate-polyacrylamide gel electrophoresis (SDS-PAGE) was done loading an equal amount of proteins per lane. Subsequently they were transferred to methanol-activated polyvinylidene difluoride (PVDF) membranes and blocked with 5% non-fat milk in TBST at room temperature for 2 h. Then the membranes were incubated with specific primary antibodies, including anti-VPS35 (1:3000, Affinity) and anti-GAPDH (1:2000, CST) in TBST overnight at 4 °C, and washed three times with TBST, afterward the membranes were left to be incubated for two hours at room temperature with secondary antibodies that matched their species. Lastly, protein visualization was carried out using the enhanced chemiluminescence (ECL) luminescence kit. For quantification, the band intensities were analyzed using ImageJ software (National Institutes of Health, USA). The relative expression level of *VPS35* was calculated by normalizing the intensity of the *VPS35* band to that of the GAPDH band in the same sample. Representative images from three independent experiments (*n* = 3) are shown.

### Silencing of VPS35 by small interfering RNA (siRNA)

Three different small interfering RNAs (siRNAs) specifically targeting human *VPS35* and a negative control (NC) siRNA were purchased from MCE (New Jersey, USA). The sequences were as follows:

si-VPS35-1: CAGAUGAGUUUGCUAAAGGAAdTdT

UUCCUUUAGCAAACUCAUCUGdTdT

si-VPS35-2: CCAGGUGGAUUCCAUAAUGAAdTdT

UUCAUUAUGGAAUCCACCUGGdTdT

si-VPS35-3: GAUGAAAUCAGCGAUUCCAAAdTdT

UUUGGAAUCGCUGAUUUCAUCdTdT

Negative control (NC): a non-targeting scrambled sequence provided by the manufacturer.

For transfection, HepG-2 and Hep3B cells were seeded into 6-well plates (2 × 10^5^ cells per well) in a complete medium for 24 hours, and subsequently transfected with *VPS35* siRNA (si-VPS35-3) or NC (at a final concentration of 100 nM) using Lipofectamine 2000 (Invitrogen, Carlsbad, CA, USA) for 6 h, then the medium was replaced with standard growth medium. At 24 h after transfection, the cells were harvested for RT-qPCR or Western blot analyses to confirm knockdown.

### Cell proliferation

The *si-VPS35* cells were seeded into 96-well plates at a density of 2000 cells per well and cultured for 0, 24 h, 48 h, 72 h, 4 days and 7 days. The cell proliferation was measured using the Cell Counting Kit-8 (C0038; Beyotime, China) following the manufacturer's protocol. Following incubation, the absorbance (OD) of the cells in the 96-well plate was measured at 450 nm with a multi-functional microplate reader (Thermo Fisher Scientific, Waltham, MA, USA). The assay was performed in triplicate and repeated in three independent experiments (*n* = 3).

### Colony-formation

After confirming successful transfection, the cells were cultured in 6-well plates with a seeding density of 2000 cells per well. The culture dishes were maintained in a humidified incubator at 37 °C with 5% CO_2_ for 7 days. Upon completion of the culture period, cells were fixed in 4% paraformaldehyde (BL539A; Biosharp, China) for 30 min. Subsequently, the cells were stained with crystal violet staining solution (C0121; Beyotime, China) for 20 min. After staining, samples were air-dried and photographed for analysis. The assay was performed in triplicate and repeated in three independent experiments (*n* = 3).

### Transwell cell migration and invasion

For the transwell cell migration assay, the successfully transfected cells (5 × 10^5^ cells) were suspended in 200 μl of serum-free medium and then added to the upper chamber (14 421 030; Corning Incorporated, USA). The lower compartment was filled with 700 μl DMEM containing 10% FBS, followed by 24-hour incubation. After incubation, the cells were fixed with 4% paraformaldehyde (PFA) for 20 min, followed by staining with crystal violet solution for 20 min. Following staining, the inner surface of the chamber was gently wiped with a moistened cotton swab to remove residual crystal violet, after which the membrane was photographed using an imaging system. For the transwell cell invasion assay, firstly 60 μl of Matrigel basement membrane matrix (356 234; Corning Incorporated, USA), diluted 1 : 8 in serum-free medium, was carefully coated onto the upper chamber surface and allowed to polymerize at 37 °C for 30 min before use. 200 μl of serum-free medium containing 5 × 10^5^ cells were added to the transwell chamber, while 700 μl of DMEM supplemented with 10% fetal bovine serum (FBS) was added to the lower chamber as a chemoattractant. After 24-hour incubation, the cells were fixed with 4% paraformaldehyde (PFA) for 15 min, and stained with 0.1% crystal violet for 20 min. The assay was performed in triplicate and repeated in three independent experiments (*n* = 3).

### Nude mouse tumor model

The mouse experiments were approved by the Biomedical Ethics Committee of the Health Science Center, Xi'an Jiaotong University, Approval Number: XJTUAE2024-2370. All procedures were conducted in accordance with the ‘Animal Research: Reporting *In Vivo* Experiments’ (ARRIVE) 2.0 guidelines. HepG2-sh-NC cells and HepG2-*sh-VPS35* cells (5 × 10^5^) were injected subcutaneously into male nude mice, respectively (*n* = 5). After injection, the mice were examined every 2 days, and tumor growth was evaluated by measuring the length and width of tumor mass (*V* = 0.5 × length × width^2^). After 15 days, nude mice were euthanized, and tumors were excised, measured, weighed, and photographed.

### Immunohistochemistry

After fixing tumor and normal tissue sections at a thickness of 10 μm in paraffin, they were subjected to dewaxing in dimethylbenzene followed by rehydration in ethanol at varying concentrations, followed by antigen repair utilizing sodium citrate solution through microwave heating at 95 °C and cooling to room temperature to extract the antigens. Then the activity of endogenous peroxidase was effectively suppressed by adding 3% H_2_O_2_ to the sections for 20 min and subsequently blocked with 5% goat normal serum for 20 min. Next, the tissue slices were incubated in a blocking solution and then exposed to 10% goat serum for one hour. The primary antibody against *VPS35* was diluted at 1 : 200 and incubated with the tissue sections all night at 4 °C, followed by the appropriate secondary antibody added to the sections to incubate them within a dark chamber. Subsequently, following the detection of immunostaining with diaminobenzidine (DAB), the sections underwent hematoxylin counterstaining, dehydration, and transparency, sealed with neutral gum, photographed, and analyzed under a microscope.

### Statistical analysis

All statistical analyses were performed using R software (version 4.0.3) and GraphPad Prism (version 8.0). The normality of data distribution was assessed using the Shapiro–Wilk test. Comparisons between two groups were performed using Student's *t*-test for normally distributed data and the Wilcoxon rank-sum test for non-normally distributed data. For comparisons among more than two groups, one-way ANOVA with the Bonferroni *post hoc* test was used for parametric data, while the Kruskal–Wallis *H* test with Bonferroni correction was used for non-parametric data. Welch's ANOVA was employed when the assumption of equal variances was violated. Correlation analysis was performed using Spearman's rank correlation coefficient. Survival analysis was conducted using the log-rank test for Kaplan–Meier curves and univariate/multivariate Cox proportional hazards regression models. The diagnostic value was assessed by receiver operating characteristic (ROC) curve analysis. All experiments were repeated in at least three independent biological replicates. All *in vivo* studies used at least five animals per group (*n* ≥ 5). Data following a normal distribution are presented as mean ± standard deviation (SD), while non-normally distributed data are presented as median and interquartile range. *P* < 0.05 was considered statistically significant.

## Results

### The expression of VPS35 in human organs and tissues

Analysis of the consensus dataset revealed predominant *VPS35* mRNA expression in bone marrow, midbrain, hypothalamus, heart muscle, skin, and spinal cord (Fig. S1A). Similarly, GTEx transcriptomic data showed abundant *VPS35* mRNA expression in the midbrain, hypothalamus, heart muscle, skin, spinal cord, and cerebral cortex (Fig. S1B). At the protein level, HPA data demonstrated that *VPS35* was predominantly expressed in bone marrow, parathyroid gland, spleen, thymus, skeletal muscle, and breast (Fig. S1C). In addition, *VPS35* protein was highly expressed in the parathyroid gland, nasopharynx, lung, stomach, duodenum, colon, rectum, gallbladder, pancreas, urinary bladder, testis, endometrium, placenta, and appendix (Fig. S1D). As shown in Fig. S1E, *VPS35* exhibits detailed expression patterns in various single-cell tissues, including the liver, testis, breast, fallopian tube, and salivary gland. Furthermore, we analyzed *VPS35* subcellular localization using immunofluorescence data from the HPA database. In A-431, U-2OS, and U-251MG cells, commonly used in cancer research due to their biological relevance and experimental tractability, *VPS35* predominantly localized to the nucleoplasm, as indicated by green fluorescence signals colocalizing with nuclear markers (Fig. S1F–S1J). These cell lines were selected for their established roles in molecular and cell biology studies, as well as their ease of culture, ensuring experimental reproducibility.

### VPS35 is highly expressed in most tumors

**Fig. 1:**
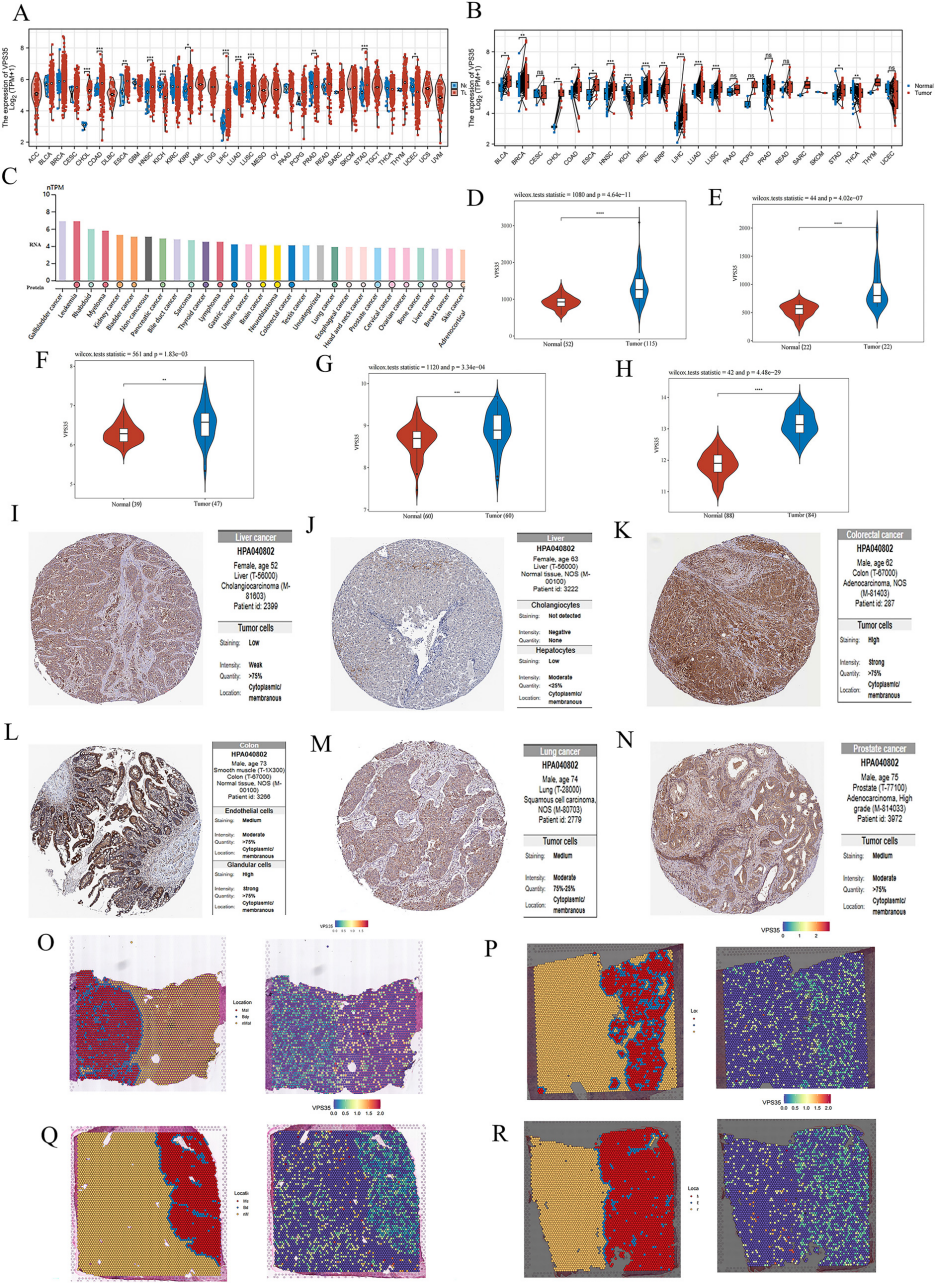
VPS35 expression in most tumors. (A and B) The mRNA expression of VPS35 in unpaired and paired pan-cancerous tissues. (C) The mRNA expression of VPS35 in different cancer cell lines. (D–F) The mRNA expression of VPS35 in LIHC based on data from three GEO databases. (G and H) The mRNA expression of VPS35 in LUAD and COAD based on data from the GEO database. (I–N) The protein expression of VPS35 in LIHC, COAD, LUAD and PRAD by IHC staining from the HPA database. (O–R)The protein expression of VPS35 in spatial transcriptomics (ns: no significance; * *p* < 0.05; ** *p* < 0.01; *** *p* < 0.001).

*VPS35* was significantly highly expressed in 12 unpaired tumor tissues, including LIHC, colon adenocarcinoma (COAD), and stomach adenocarcinoma (STAD) (Fig. 1A). Moreover, it showed significant upregulation in 14 paired tumor tissues compared with adjacent normal controls, such as LIHC, lung squamous cell carcinoma (LUSC), and kidney renal clear cell carcinoma (KIRC) (Fig. 1B). Additionally, *VPS35* exhibited broad-spectrum expression across pan-cancer cell lines, with particularly high levels in gallbladder cancer and leukemia (Fig. 1C). Furthermore, we validated the significant upregulation of *VPS35* in LIHC through three independent GEO datasets (GSE76427, GSE84598, and GSE45267) (Fig. 1D–F). Consistently, elevated *VPS35* expression was observed in both LUAD (GSE19804 dataset) and COAD (GSE37182 dataset) (Fig. 1G and H). To further validate *VPS35* protein expression levels, we retrieved IHC staining images from the HPA database. These images demonstrated elevated *VPS35* expression in COAD, LIHC, LUAD (lung adenocarcinoma), and PRAD (prostate adenocarcinoma) tissues compared to normal controls (Fig. 1I–N). In the spatial transcriptome samples of four LIHC samples, *VPS35* expression was significantly upregulated in tumors (Fig. 1O–R).

### The diagnostic value of VPS35 in pan-cancer

**Fig. 2:**
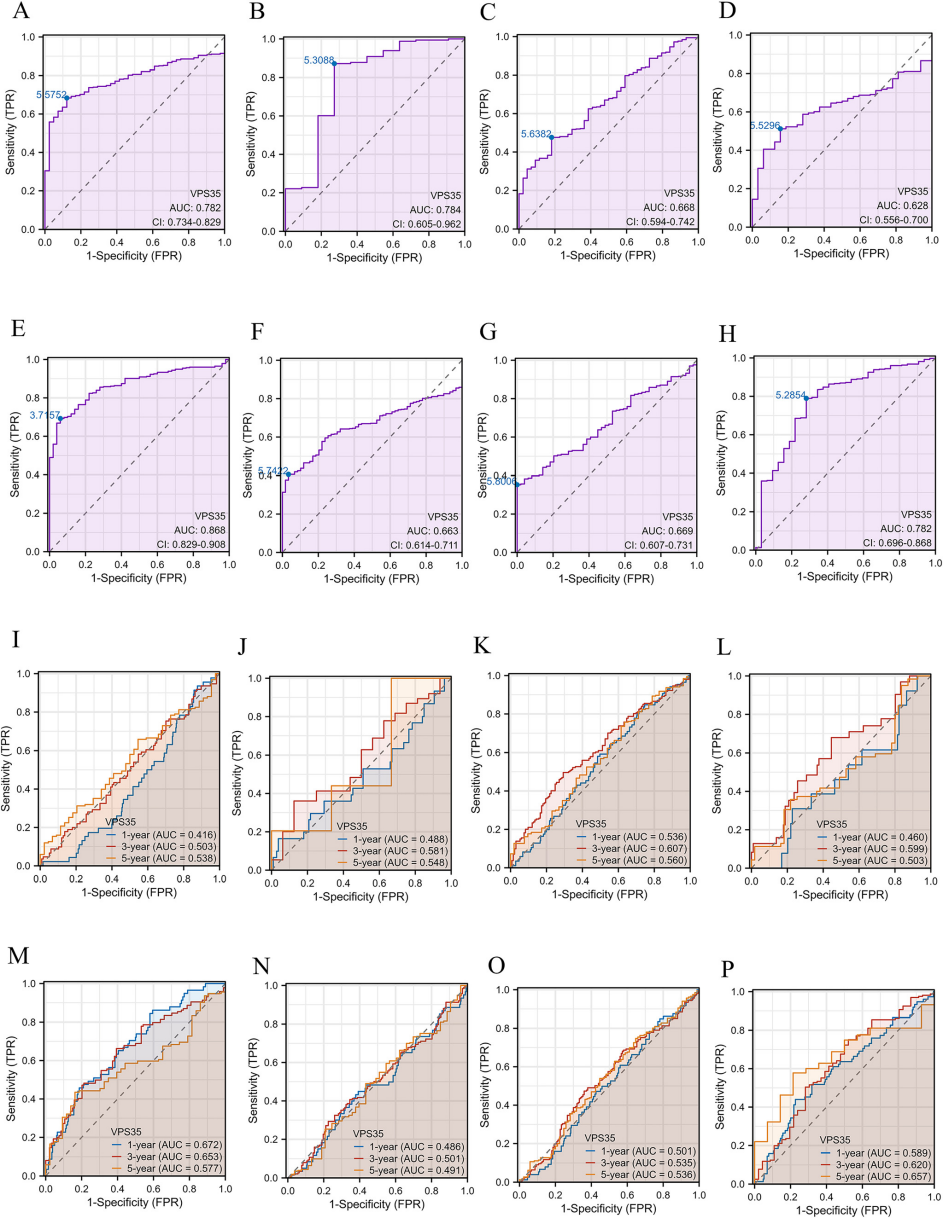
The diagnostic values of VPS35 in pan-cancer. (A–H) ROC curves were used to predict the diagnostic value of VPS35 in pan-cancer. (I–P) Time-dependent ROC curves were used to predict the diagnostic value of VPS35 in pan-cancer.

As shown in Fig. 2A–H, VPS35 showed moderate diagnostic accuracy for HNSC (AUC = 0.668, 95% CI: 0.594–0.742), KIRP (AUC = 0.628, 95% CI: 0.556–0.700), LUAD (AUC = 0.663, 95% CI: 0.614–0.711) and LUSC (AUC = 0.669, 95% CI: 0.607–0.731). In addition, *VPS35* has a good diagnostic value for COAD (AUC = 0.782, 95% CI: 0.734–0.829), ESCA (AUC = 0.784, 95% CI: 0.605–0.962), STAD (AUC = 0.782, 95% CI: 0.696–0.868) and LIHC (AUC = 0.868, 95% CI: 0.829–0.908). Furthermore, time-dependent ROC analysis demonstrated that *VPS35* showed high predictive values for 1-, 3-, and 5-year OS in COAD, ESCA, HNSC, KIRP, LIHC, LUAD, LUSC, and STAD (Fig. 2I–P).

### The prognostic value of VPS35 in pan-cancer

**Fig. 3:**
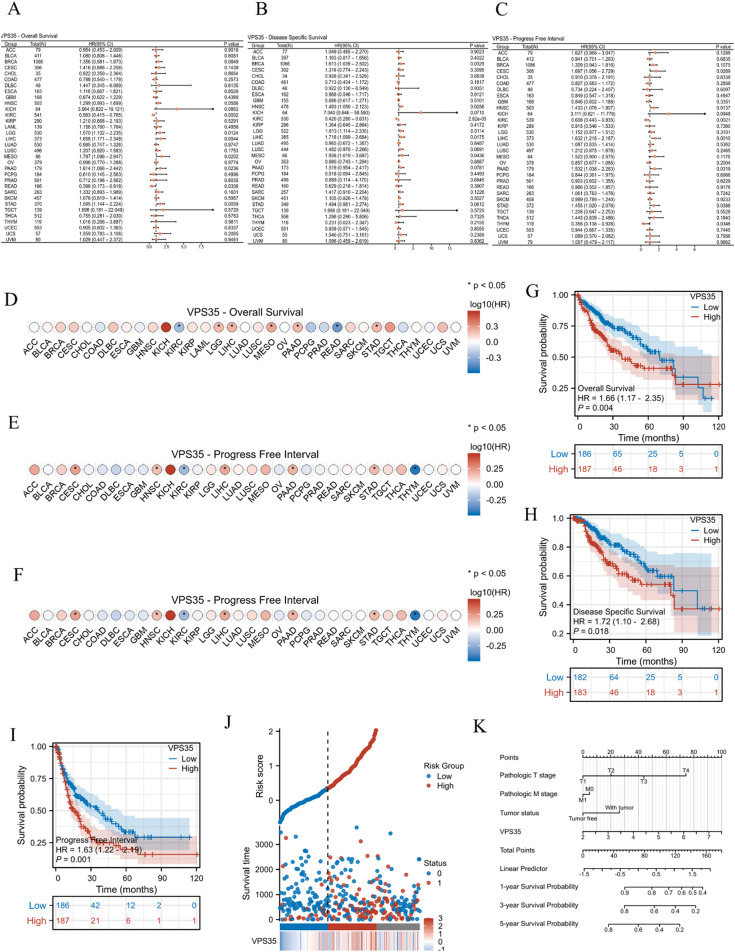
The prognostic value of VPS35 in pan-cancer. (A–C) The forest plot shows the univariate Cox regression analysis results of VPS35 on OS, DSS, and PFI in TCGA pan-cancer. (D–F) The prognostic heatmap shows the prognostic value of VPS35 on OS, DSS, and PFI in TCGA pan-cancer. (G–I) Kaplan–Meier survival analysis the prognostic value of VPS35 for OS, PFI, and DSS of LIHC in the TCGA A cohort. (J) VPS35 expression distribution and survival status. 0: dead; 1: alive. (K) The prognostic nomogram of VPS35 expression levels in LIHC.

The basic information of all patients included in this study from the TCGA-LIHC cohort is presented in Table 1. To investigate the prognostic value of *VPS35*, we performed univariate Cox regression analysis to evaluate *VPS35* expression with OS, DSS, and PFI in pan-cancer. The forest map (Fig. 3A–C) and prognostic heatmap (Fig. 3D–F) show the prognostic value of *VPS35* in a variety of cancer types. To further clarify the prognostic value of *VPS35* in LIHC, we performed survival analysis using Kaplan–Meier curves. The results demonstrated that a higher expression level of *VPS35* was associated with poorer prognosis in LIHC patients (Fig. 3G–I). We further evaluated the prognostic value of *VPS35* in different LIHC subgroups, and the results consistently demonstrated its significant prognostic relevance across all subsets, such as tumor status (HR = 2.15, 95% CI: 1.15–4.00, *P* = 0.016), gender (HR = 1.74, 95% CI: 1.11–2.74, *P* = 0.017) and prothrombin time (HR = 1.75, 95% CI: 1.01–3.04, *P* = 0.047) (Fig. S2A–S2J). We generated a risk factor plot using R software to illustrate the association between *VPS35* expression and survival outcomes in LIHC patients. In this visualization, blue dots represent alive patients while red dots indicate deceased cases. Notably, the density of red dots increased progressively with higher risk scores, demonstrating that elevated *VPS35* expression correlates with significantly increased mortality risk in LIHC (Fig. 3J).

**Table 1:** Baseline data table of VPS35 in HCC

Characteristics	Low expression of VPS35	High expression of VPS35	*P* value
*n*	187	187	
Age, *n* (%)			0.132
<=60	81 (21.7%)	96 (25.7%)	
>60	105 (28.2%)	91 (24.4%)	
Gender, *n* (%)			0.320
Female	56 (15%)	65 (17.4%)	
Male	131 (35%)	122 (32.6%)	
Weight, *n* (%)			**0.007**
<=70	81 (23.4%)	103 (29.8%)	
>70	95 (27.5%)	67 (19.4%)	
Height, *n* (%)			0.800
<170	102 (29.9%)	99 (29%)	
>=170	73 (21.4%)	67 (19.6%)	
BMI, *n* (%)			0.086
<=25	83 (24.6%)	94 (27.9%)	
>25	90 (26.7%)	70 (20.8%)	
Histologic grade, *n* (%)			**0.034**
G1 & G2	126 (34.1%)	107 (29%)	
G3 & G4	58 (15.7%)	78 (21.1%)	
Residual tumor, *n* (%)			**0.040**
R0	172 (49.9%)	155 (44.9%)	
R1 & R2	5 (1.4%)	13 (3.8%)	
AFP (ng ml^−1^), *n* (%)			0.171
<=400	120 (42.9%)	95 (33.9%)	
>400	30 (10.7%)	35 (12.5%)	
Albumin (g dl^−1^), *n* (%)			0.268
<3.5	33 (11%)	36 (12%)	
>=3.5	128 (42.7%)	103 (34.3%)	
Prothrombin time, *n* (%)			0.694
<=4	107 (36%)	101 (34%)	
>4	48 (16.2%)	41 (13.8%)	
Child-Pugh grade, *n* (%)			**0.034**
A	131 (54.4%)	88 (36.5%)	
B & C	8 (3.3%)	14 (5.8%)	
Fibrosis ishak score, *n* (%)			0.474
0	44 (20.5%)	31 (14.4%)	
1/2 & 3/4 & 5 & 6	75 (34.9%)	65 (30.2%)	
Vascular invasion, *n* (%)			0.080
No	116 (36.5%)	92 (28.9%)	
Yes	50 (15.7%)	60 (18.9%)	
Adjacent hepatic tissue inflammation, *n* (%)			0.560
None	59 (24.9%)	59 (24.9%)	
Mild & severe	64 (27%)	55 (23.2%)	
Tumor status, *n* (%)			0.081
Tumor free	110 (31%)	92 (25.9%)	
With tumor	69 (19.4%)	84 (23.7%)	
Pathologic T stage, *n* (%)			**0.013**
T1 & T2	149 (40.2%)	129 (34.8%)	
T3 & T4	36 (9.7%)	57 (15.4%)	
Pathologic N stage, *n* (%)			0.147
N0	125 (48.4%)	129 (50%)	
N1	0 (0%)	4 (1.6%)	
Pathologic M stage, *n* (%)			0.553
M0	127 (46.7%)	141 (51.8%)	
M1	3 (1.1%)	1 (0.4%)	
Pathologic stage, *n* (%)			**0.001**
Stage I & stage II	143 (40.9%)	117 (33.4%)	
Stage III & stage IV	32 (9.1%)	58 (16.6%)	
Race, *n* (%)			0.267
Asian	79 (21.8%)	81 (22.4%)	
Black or African American	5 (1.4%)	12 (3.3%)	
White	92 (25.4%)	93 (25.7%)	

In univariate Cox regression analysis, variables with *P* < 0.05 were identified as risk factors, whereas those retaining statistical significance (*P* < 0.05) in multivariate Cox regression were considered independent risk factors. Notably, univariate Cox regression analysis indicated that the pathologic stage (HR = 2.50, 95% CI: 1.73–3.63, *P* < 0.001), pathologic T stage (HR = 2.60, 95% CI: 1.83–3.70, *P* < 0.001), pathologic M stage (HR = 4.08, 95% CI: 1.28–12.97, *P* = 0.017), tumor status (HR = 2.32, 95% CI: 1.59–3.38, *P* < 0.001) and *VPS35* expression (HR = 1.64, 95% CI: 1.28–2.10, *P* < 0.001) were significantly associated with shorter OS in LIHC. Furthermore, multivariate Cox regression found that tumor status (HR = 1.83, 95% CI: 1.14–2.93, *P* = 0.013) and VPS35 expression (HR = 1.50, 95% CI: 1.07–2.11, *P* = 0.019) were independent risk factors for OS in LIHC (Table 2). Based on the Cox regression analysis results, we further generated a prognostic nomogram for LIHC incorporating *VPS35* expression levels (Fig. 3K).

**Table 2:** Correlation of the VPS35 expression level with clinicopathological features in TCGA-LIHC

Characteristics	Total (N)	Univariate analysis		Multivariate analysis	
		Hazard ratio (95% CI)	*P* value	Hazard ratio (95% CI)	*P* value
Pathologic T stage	370				
T1 & T2	277	Reference		Reference	
T3 & T4	93	2.598 (1.826–3.697)	**<0.001**	2.189 (0.289–16.580)	0.448
Pathologic M stage	272				
M0	268	Reference		Reference	
M1	4	4.077 (1.281–12.973)	**0.017**	1.593 (0.374–6.794)	0.529
Pathologic stage	349				
Stage I & stage II	259	Reference		Reference	
Stage III & stage IV	90	2.504 (1.727–3.631)	**<0.001**	1.050 (0.138–7.961)	0.962
Tumor status	354				
Tumor free	202	Reference		Reference	
With tumor	152	2.317 (1.590–3.376)	**<0.001**	1.826 (1.138–2.929)	**0.013**
Gender	373				
Female	121	Reference			
Male	252	0.793 (0.557–1.130)	0.200		
Race	361				
Asian	159	Reference			
Black or African American	17	1.585 (0.675–3.725)	0.290		
White	185	1.323 (0.909–1.928)	0.144		
Age	373				
<=60	177	Reference			
>60	196	1.205 (0.850–1.708)	0.295		
BMI	336				
<=25	177	Reference			
>25	159	0.798 (0.550–1.158)	0.235		
Residual tumor	344				
R0	326	Reference			
R1 & R2	18	1.604 (0.812–3.169)	0.174		
Histologic grade	368				
G1 & G2	233	Reference			
G3 & G4	135	1.091 (0.761–1.564)	0.636		
AFP (ng ml^−1^)	279				
<=400	215	Reference			
>400	64	1.075 (0.658–1.759)	0.772		
Albumin (g dl^−1^)	299				
<3.5	69	Reference			
>=3.5	230	0.897 (0.549–1.464)	0.662		
Prothrombin time	296				
<=4	207	Reference			
>4	89	1.335 (0.881–2.023)	0.174		
Vascular invasion	317				
No	208	Reference			
Yes	109	1.344 (0.887–2.035)	0.163		
VPS35	373	1.638 (1.281–2.095)	**<0.001**	1.502 (1.070–2.110)	**0.019**

### The correlation between VPS35 expression and clinicopathological characteristics in LIHC

**Fig. 4:**
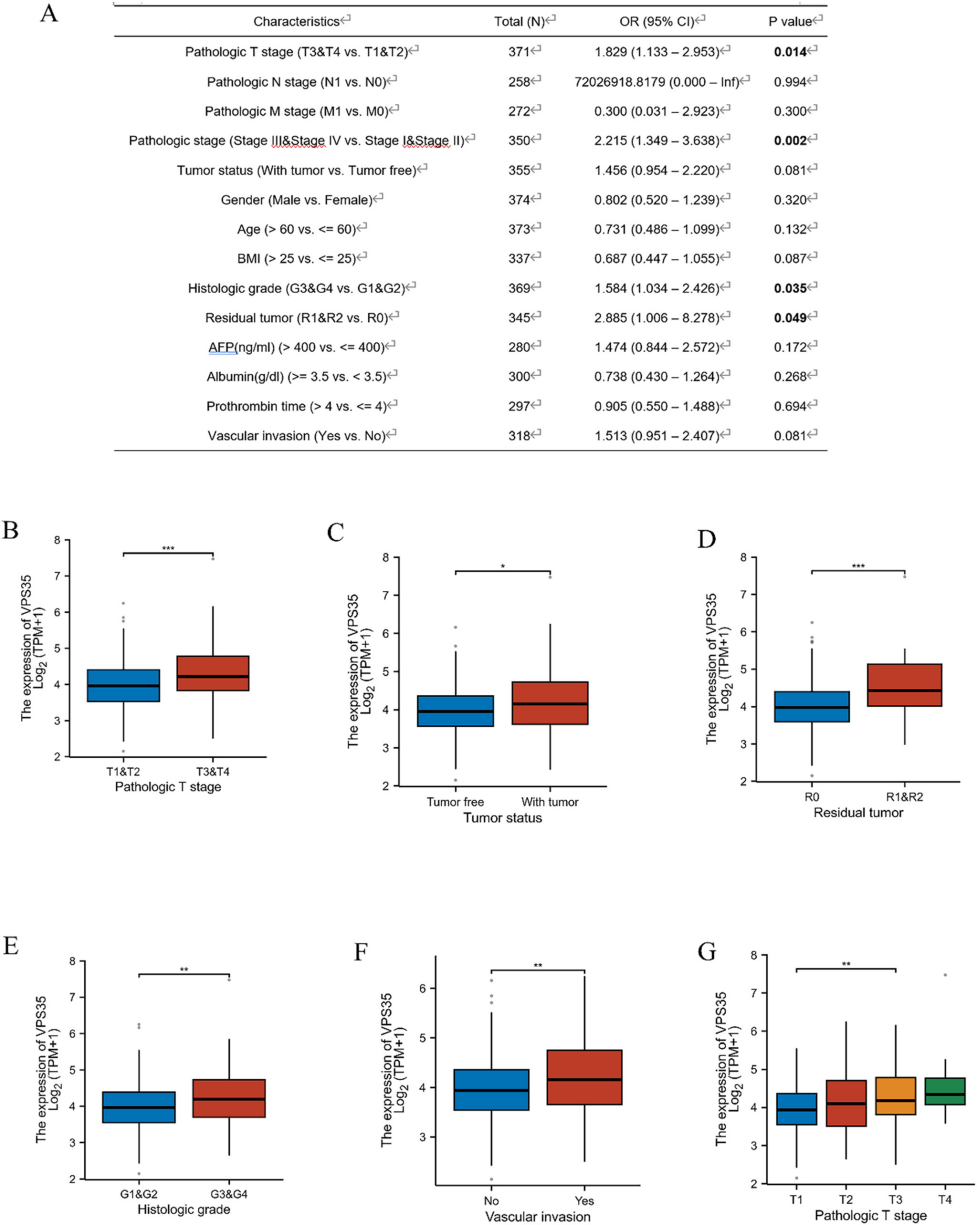
The correlation between VPS35 expression and clinicopathological characteristics in LIHC. (A) Relationship between VPS35 expression and clinicopathological features in TCGA-HCC. (B–G) The correlations of VPS35 expression with pathologic T stage, residual tumor, histologic grade, vascular invasion in LIHC. (ns: no significance; * *p* < 0.05; ** *p* < 0.01; *** *p* < 0.001).

Logistic regression analysis found that *VPS35* expression was positively correlated with the pathologic T stage (OR = 1.97, 95% CI: 1.13–2.95, *P* = 0.014), pathologic stage (OR = 2.22, 95% CI: 1.35–3.64, *P* = 0.002), histologic grade (OR = 1.58, 95% CI: 1.03–2.43, *P* = 0.035) and residual tumor (OR = 2.89, 95% CI: 1.01–8.28, *P* = 0.049) (Fig. 4A). In addition, subgroup analysis shows that high expression of *VPS35* was associated with tumor status, residual tumor, histologic grade, vascular invasion, pathologic stage and pathologic T stage (Fig. 4B–G).

### The gene mutation of VPS35 in pan-cancer

**Fig. 5:**
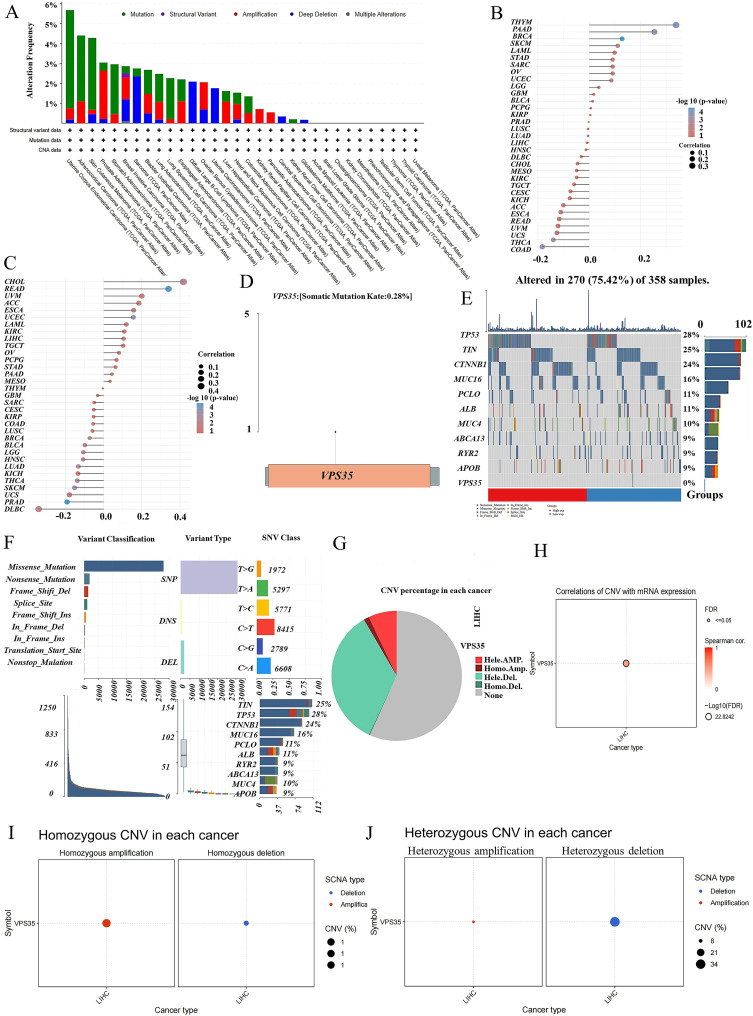
The gene mutation of VPS35 in pan-cancer. (A) The genetic alteration frequencies of VPS35 in pan-cancer. (B and C) The correlation between VPS35 expression and TMB as well as MSI according to the TCGA database. (D) Somatic mutation landscape analysis of VPS35 in LIHC. (E) The top ten genes with the highest mutation frequency of VPS35. (F) Variant classification analysis of VPS35 in LIHC. (G) Copy number variations of VPS35 in LIHC. (H) The correlation with VPS35 CNV status and its mRNA expression levels. (I) The proportions of homozygous deletions and homozygous amplifications. (J) The proportions of heterozygous deletions and heterozygous amplifications.

Analysis of *VPS35* mutation frequency across pan-cancer revealed that uterine corpus endometrial carcinoma (5.7%), adrenocortical carcinoma (4.3%), and skin cutaneous melanoma (4.2%) exhibited the highest mutation rates, with structural variants (SV) being the most common mutation type of *VPS35* (Fig. 5A). TMB and MSI correlation analyses showed that *VPS35* expression was positively correlated with TMB in 14 cancer types and with MSI in 15 cancer types (Fig. 5B and C). Somatic mutation landscape analysis in LIHC demonstrated a *VPS35* mutation rate of 0.28% (Fig. 5D). The top 10 most frequently mutated genes in the *VPS35* high- and low-expression groups are shown in Fig. 5E. Variant classification indicated that missense mutations were the predominant alteration type in LIHC (Fig. 5F). Further investigation of copy number variations (CNV) in LIHC revealed that *VPS35* exhibited both homozygous and heterozygous CNVs (Fig. 5G). *VPS35* CNV status showed a strong positive correlation with its mRNA expression levels (Fig. 5H). The proportions of homozygous deletions and homozygous amplifications were 1% each (Fig. 5I), while heterozygous deletions accounted for 21% and heterozygous amplifications for 8% (Fig. 5J).

### Differentially expressed gene and enrichment analysis

**Fig. 6:**
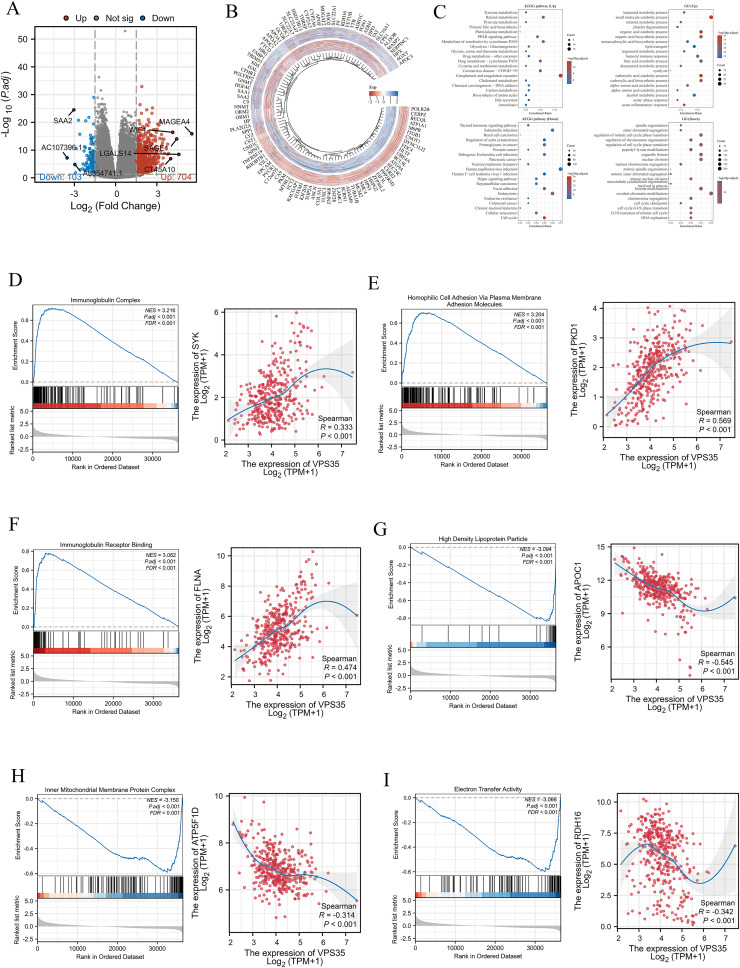
Differentially expressed gene (DEG) and functional enrichment analysis. (A and B) Volcano plots of the identified DEGs. (C) GO and KEGG enrichment analysis of DEGs related to VPS35. (D–I) GSEA for the VPS35 high-expression group and correlation of VPS35 with core genes in the identified pathway.

As shown in the volcano plot, 704 upregulated and 103 downregulated genes were identified in LIHC (|log 2FC| > 1.5, FDR < 0.05) (Fig. 6A and B). These DEGs were further subjected to GO and GSEA. GO enrichment analysis revealed that the upregulated DEGs were significantly enriched in the terpenoid metabolic process, small molecule catabolic process, retinoid metabolic process, and so on. And downregulated DEGs were enriched in spindle organization, sister chromatid segregation, peptidyl-lysine modification, and so on. In addition, we identified that upregulated DEGs in LIHC were significantly enriched in the complement and coagulation cascades, retinol metabolism, metabolism of xenobiotics by cytochrome P450, and drug metabolism-cytochrome P450, while downregulated DEGs in LIHC were significantly enriched in the proteoglycans in cancer, human T-cell leukemia virus 1 infection, regulation of actin cytoskeleton, and the cell cycle (Fig. 6C). Furthermore, GSEA revealed that high *VPS35* in LIHC was enriched in immunoglobulin complex (NES = 3.216, FDR < 0.001), homophilic cell adhesion *via* plasma membrane adhesion molecules (NES = 3.204, FDR < 0.001) and immunoglobulin receptor binding (NES = 3.082, FDR < 0.001), while low *VPS35* in LIHC was enriched in high density lipoprotein particles (NES = −3.094, FDR < 0.001), inner mitochondrial membrane protein complex (NES = −3.150, FDR < 0.001) and electron transfer activity (NES = −3.066, FDR < 0.001). *VPS35* expression was positively significantly associated with homophilic cell adhesion *via* plasma membrane adhesion molecules (rs = 0.569, *P* < 0.001), immunoglobulin complex (rs = 0.333, *P* < 0.001), and immunoglobulin receptor binding (rs = 0.474, *P* < 0.001). Conversely, *VPS35* expression was negatively significantly associated with electron transfer activity (rs = −0.342, *P* < 0.001), high-density lipoprotein particles (rs = −0.545, *P* < 0.001), and inner mitochondrial membrane protein complex (rs = −0.314, *P* < 0.001) (Fig. 6D–I).

### VPS35-related genes and PPI network

**Fig. 7:**
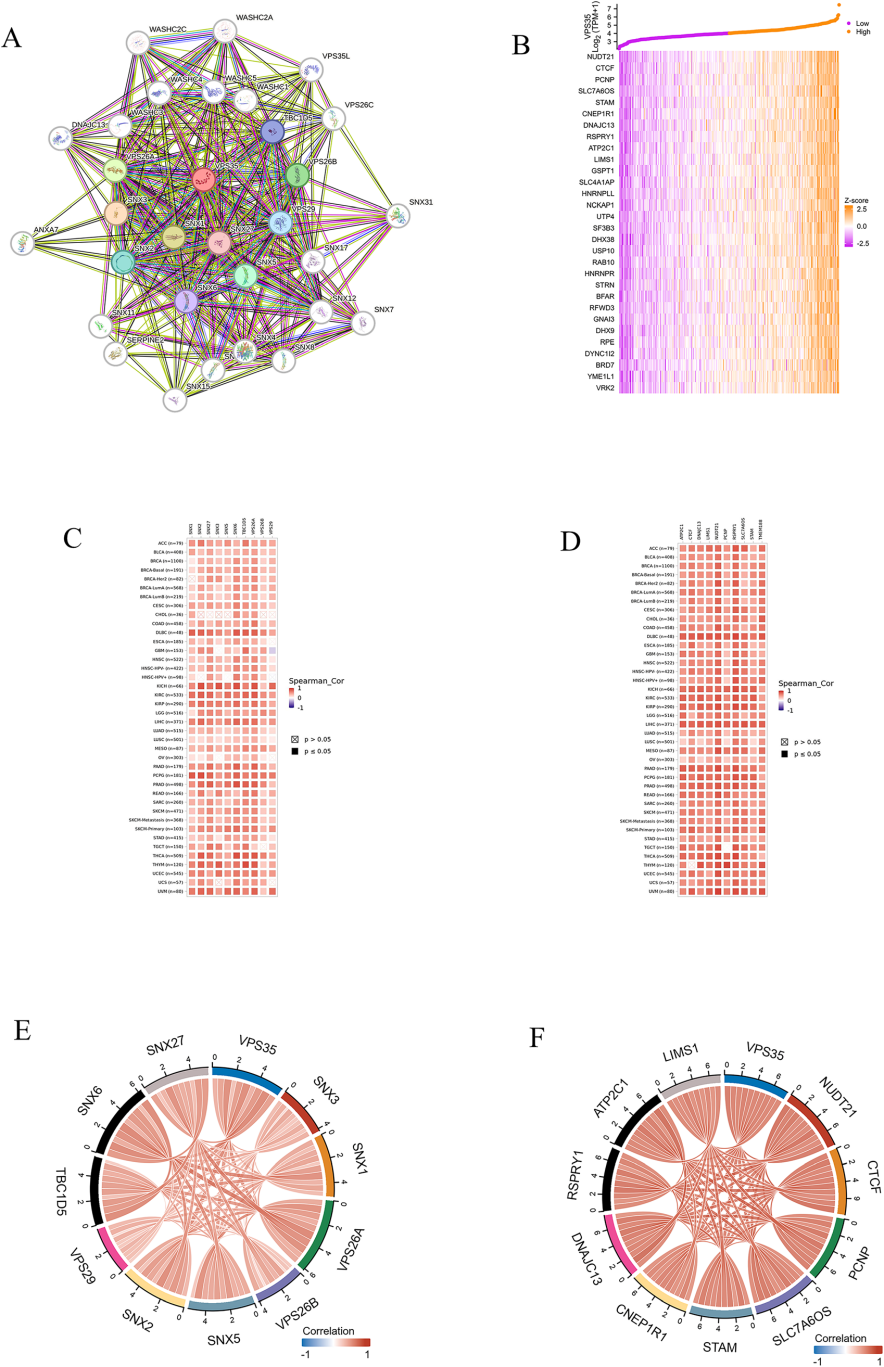
VPS35-related genes and PPI network. (A) The top 30 VPS35-related proteins *via* PPI network analysis. (B) Heatmap of top 30 co-expressed genes of VPS35 in LIHC. (C) Heat map of the top 10 correlations in the co-expression network in pan-cancer. (D) Heat map of the top 10 correlations in the PPI network in pan-cancer. (E) Heat map of the top 10 correlations in the co-expression network in LIHC. (F) Heat map of the top 10 correlations in the PPI network in LIHC.

RNA sequencing data obtained from the TCGA database were used to identify *VPS35*-related genes. To characterize the PPI network of *VPS35*, we employed the STRING database and generated a visualization of its 30 highest-scoring interactors, as shown in Fig. 7A. The heatmap (Fig. 7B) displays the 30 genes showing the highest correlation with *VPS35*, as identified through R-based analysis. In addition, we selected the top 10 genes with the highest interaction score and the top 10 genes in the correlation analysis results. Furthermore, the co-expression of these genes with *VPS35* in pan-cancer was analyzed by TlMER2, and the results showed that these 20 genes were co-expressed with *VPS35* in most cancers (Fig. 7C and D). Focused analysis in LIHC demonstrated strong intercorrelations among these selected genes, suggesting potential functional synergy in hepatocellular carcinoma pathogenesis (Fig. 7E and F).

### Prognostic value of VPS35-related genes in LIHC

**Fig. 8:**
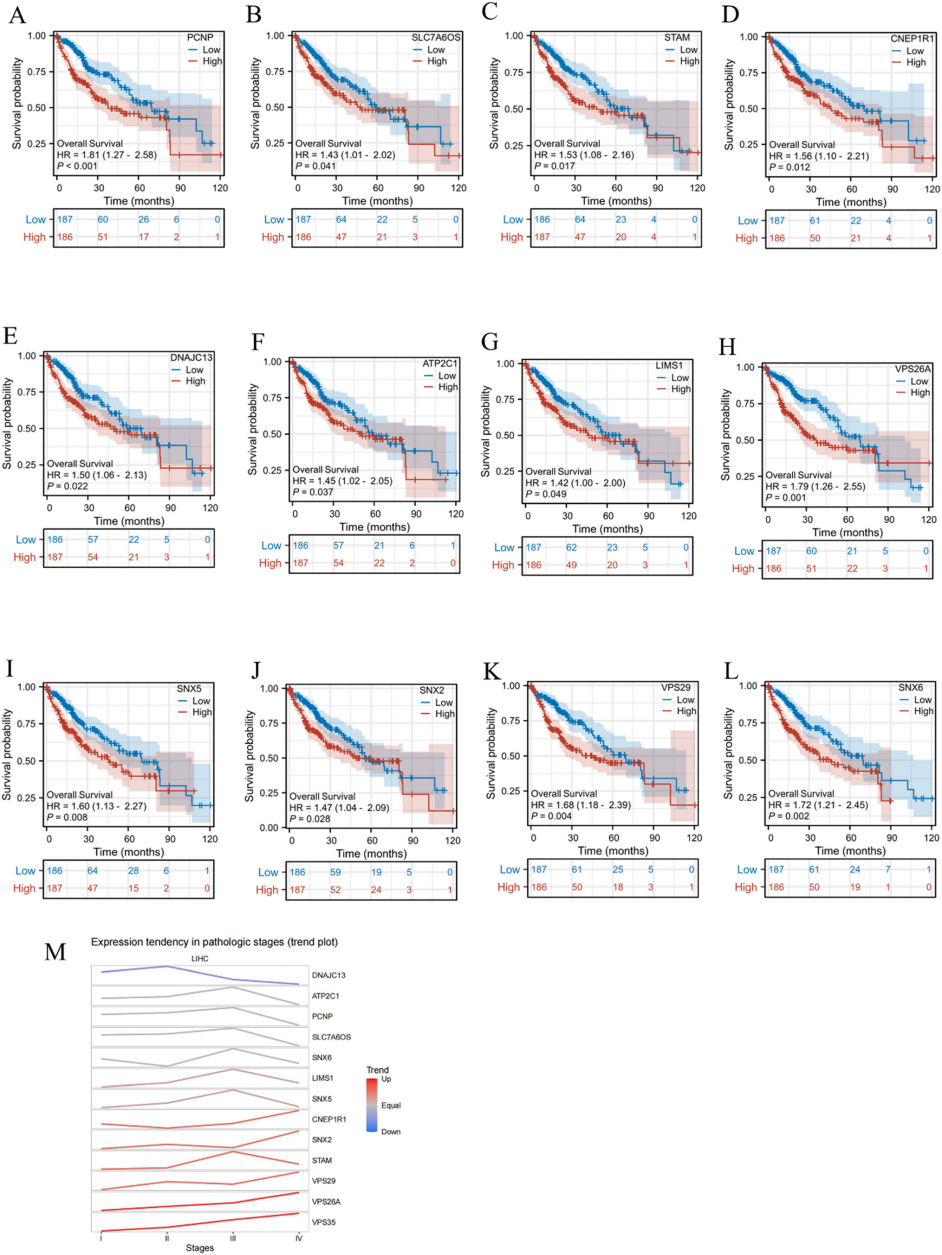
Prognostic value of VPS35-related genes in LIHC. (A–L) Kaplan–Meier survival curves for 12 genes associated with prognosis in LIHC. (M) Expression levels of the 12 prognostic genes across different clinical stages of LIHC.

Kaplan–Meier survival analysis was performed on the top 10 genes most highly co-expressed with *VPS35* and the top 10 genes with the strongest PPI interactions with *VPS35*. Our analysis revealed that a total of 12 genes were all significantly correlated with poor prognosis in LIHC, including PCNP (HR = 1.81, 95% CI. 1.27–2.58, *P* < 0.001), SLC76OS (HR = 1.43, 95% CI. 1.01–2.02, *P* = 0.041), STAM (HR = 1.53, 95% CI. 1.08–2.16, *P* = 0.017), CNEP1R1 (HR = 1.56, 95% CI. 1.10–2.21, *P* = 0.012), DNAJC13 (HR = 1.50, 95% CI. 1.06–2.13, *P* = 0.022), ATP2C1 (HR = 1.45, 95% CI. 1.02–2.05, *P* = 0.037), L1MS1 (HR = 1.42, 95% CI. 1.00–2.00, *P* = 0.049), VPS26A (HR = 1.79, 95% CI. 1.26–2.55, *P* = 0.001), SNX5 (HR = 1.60, 95% CI. 1.13–2.27, *P* = 0.008), SNX2 (HR = 1.47, 95% CI. 1.04–2.09, *P* = 0.028), VPS29 (HR = 1.68, 95% CI. 1.18–2.39, *P* = 0.004), and SNX6 (HR = 1.72, 95% CI. 1.21–2.45, *P* = 0.002) (Fig. 8A–L). Furthermore, we evaluated the expression of a total of 12 genes across different LIHC stages. The results demonstrated that *VPS26A* exhibited progressively increased expression levels with disease progression (Fig. 8M).

### Correlation between VPS35 expression and TIME in pan-cancer

**Fig. 9:**
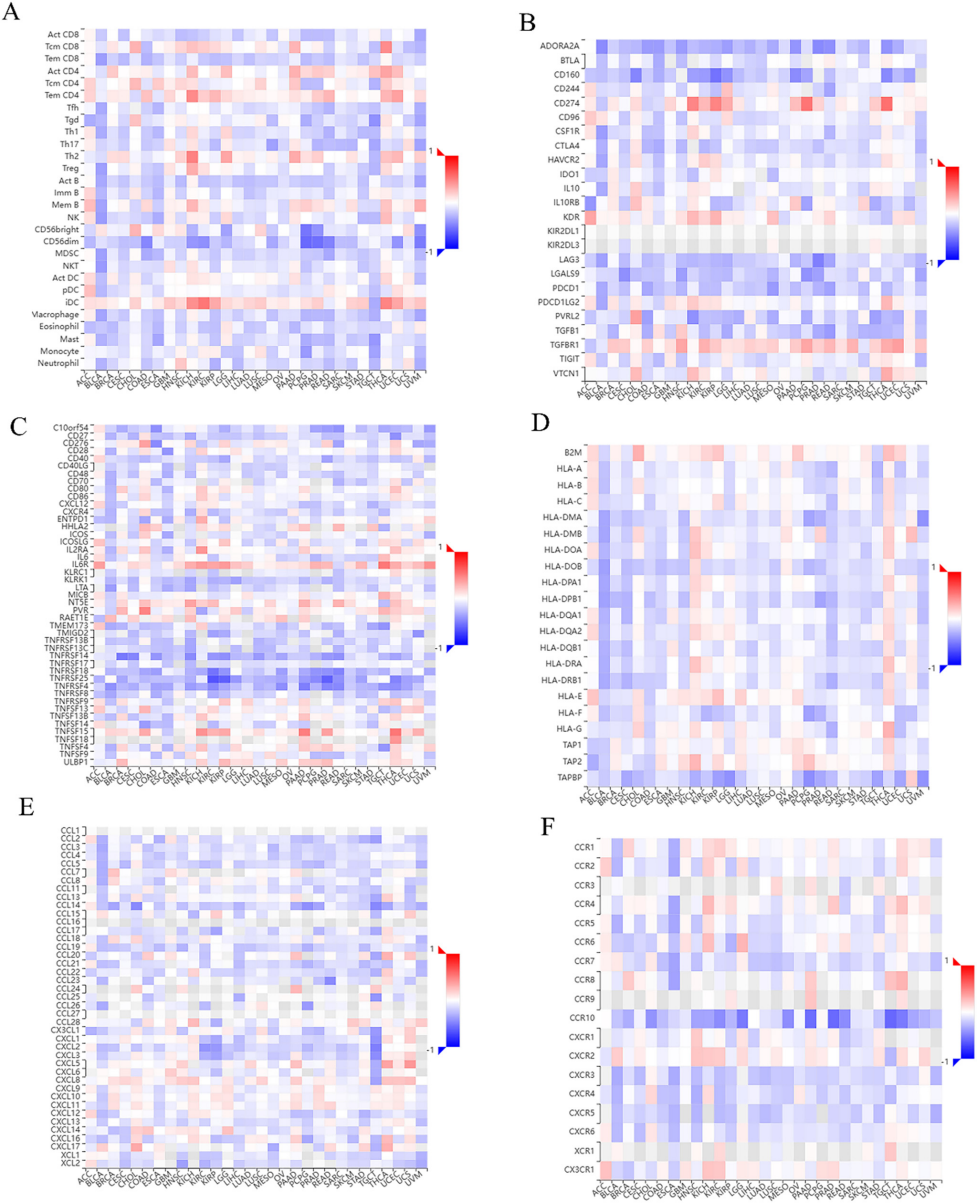
Correlation analysis of VPS35-related genes with TIME in pan-cancer. (A) Heat map of the correlation between VPS35-related genes and immune cell infiltrates. (B) Heat map of the correlation between VPS35-related genes and immunostimulators. (C) Heat map of the correlation between VPS35-related genes and immune inhibitors. (D) Heat map of the correlation between VPS35-related genes and MHC molecules. (E) Heat map of the correlation between VPS35-related genes and chemokines. (F) Heat map of the correlation between VPS35-related genes and chemokine receptors.

TISIDB analysis revealed that *VPS35* expression showed a significant positive correlation with TILs abundance (*e.g.*, Tem CD4, Th2, iDC) in KICH, while a negative correlation with CD56bright and CD56dim NK cell populations in PCPG (Fig. 9A). In addition, high *VPS35* expression was associated with upregulated immunoinhibitors (*e.g.*, *CD274*, *VTCN1*, *TGFBR1*) and immunostimulators (*e.g.*, *TNFSF15*, *TNFSF18*, *IL6R*) in THCA (Fig. 9B and C). Moreover, Fig. 9D showed that *VPS35* expression was negatively associated with TAPBP in pan-cancer, while positively correlated with B2M in the majority of human cancers, such as THCA, UCEC, and PAAD. Furthermore, *VPS35* expression was positively associated with chemokines (*e.g.*, CCL25, CCL14, CCL21) and receptors (*e.g.*, CCR7, CXCR1, CXCR3) in LIHC (Fig. 9E–F).

### The single-cell expression of VPS35 in LIHC

**Fig. 10:**
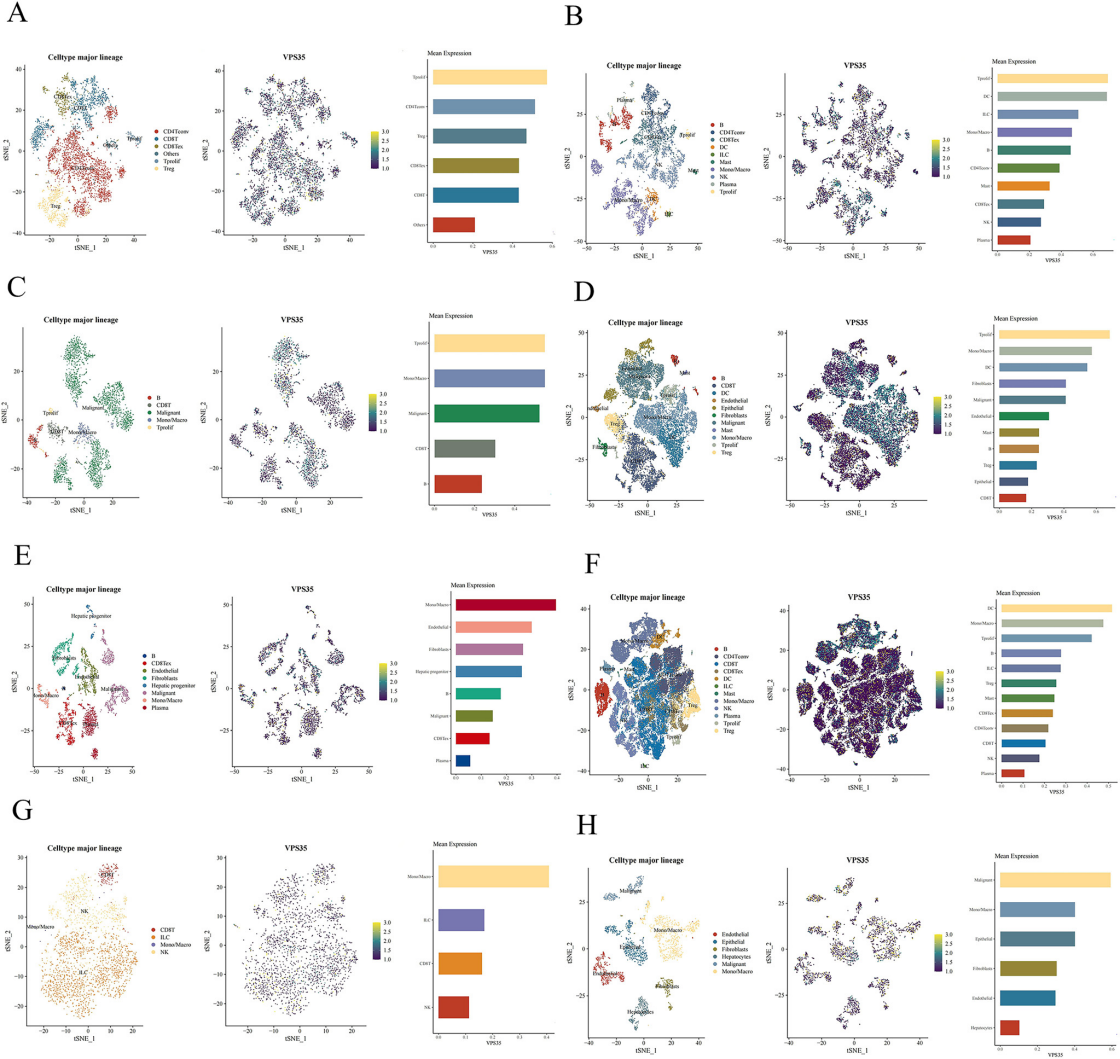
The single-cell expression of VPS35 in LIHC. (A–H) A single-cell-level global view of immune cell and VPS35 expression sites is shown in eight LIHC datasets.

We assessed the association between immune cell distribution and *VPS35* expression levels at single-cell resolution using scRNA-seq data from 8 independent LIHC datasets in the TISCH2 database. As shown in clustered plots, analysis of scRNA-seq showed that VPS35 expression patterns varied among immune cell types, suggesting its association with the LIHC immune landscape. For instance, *VPS35* exhibited the highest expression levels in proliferating T cell (Tprolif) clusters across the GSE98638, GSE140228_Smart-seq2, GSE146115, and GSE166635 datasets. Similarly, in the GSE125449 and GSE179795 datasets, *VPS35* expression was most abundant in monocytes/macrophages (mono/macro). Furthermore, *VPS35* showed peak expression in dendritic cells (DC) (GSE140228_10x) and malignant cells (GSE146409), respectively (Fig. 10A–H).

### The immunotherapy and chemotherapy response analysis of VPS35

**Fig. 11:**
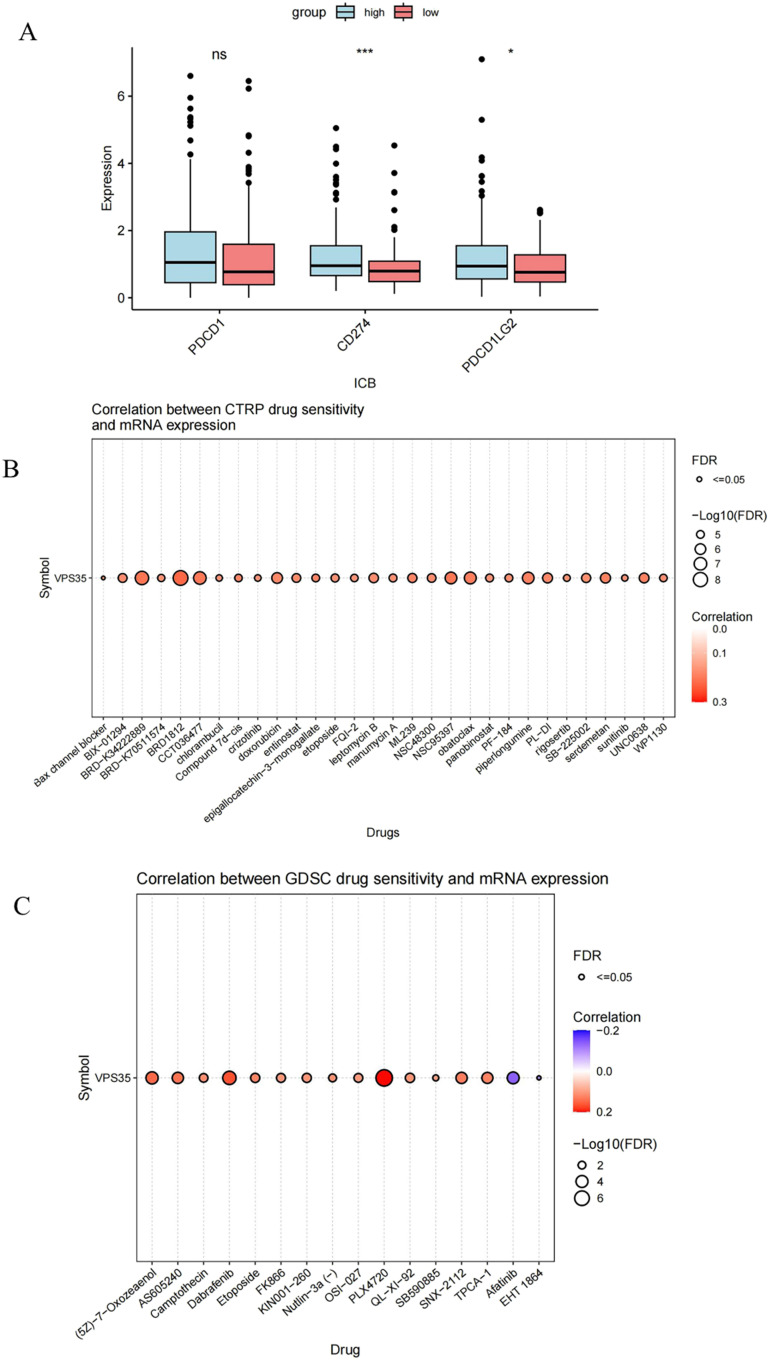
The immunotherapy and chemotherapy response analysis of VPS35. (A) The predictive responses of ICI-related markers between VPS35-high and VPS35-low groups. (B and C) The predictive value of VPS35 for GDSC and CTRP drug therapy in pan-cancer (ns: no significance; * *p* < 0.05; ** *p* < 0.01; *** *p* < 0.001).

To assess the clinical relevance of *VPS35* in immunotherapy, we compared ICI responses between *VPS35*-high and *VPS35*-low samples. Using the TIDE algorithm, we predicted ICI-related markers (PD-1/PDCD1, PD-L1/CD274, and PD-L2/PDCD1LG2) and observed significantly lower TIDE scores for PD-L1 in the VPS35-low group, suggesting that low *VPS35* expression may be associated with improved ICI response in LIHC (Fig. 11A). Analysis of the CTRP and GDSC databases revealed that the *VPS35* expression level was correlated with sensitivity to multiple chemotherapeutic agents (*e.g.*, doxorubicin, BRD1812, PLX4720) across pan-cancer (Fig. 11B and C). Further investigation in LIHC demonstrated that the *VPS35* high-expression group exhibited lower IC_50_ values for axitinib, carmustine, cediranib, and ibrutinib drugs, suggesting enhanced drug sensitivity in these tumors. Conversely, the *VPS35* low-expression group showed higher IC_50_ values for cisplatin, dabragenib, entinostat, erintinib, irinotecan, and oxaliplatin drugs, indicating potential drug resistance when *VPS35* is downregulated (Fig. S3A).

### Correlation of VPS35 with ferroptosis and m6A-related genes in LIHC

**Fig. 12:**
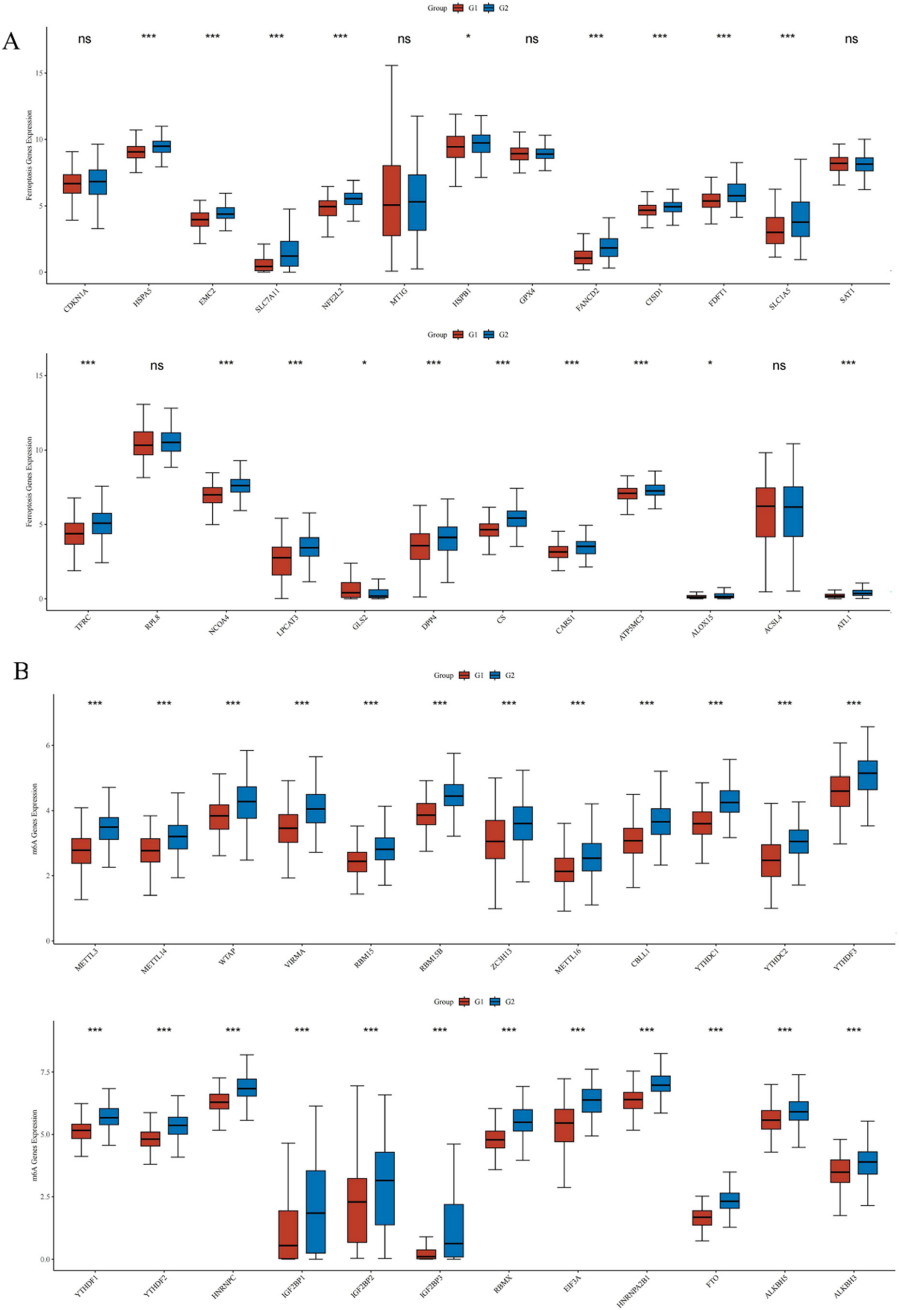
Correlation of VPS35 with ferroptosis and m6A-related genes in LIHC. (A) Correlation of VPS35 with ferroptosis-related genes in LIHC. (B) Correlation of VPS35 with m6A-related genes in LIHC (ns: no significance; * *p* < 0.05; ** *p* < 0.01; *** *p* < 0.001).

Ferroptosis-related genes were significantly upregulated in LIHC with high *VPS35* expression, including HSPA5, EMC2, SLC7A11, NFE2L2, HSPB1, FANCD2, CISD1, FDFT1, SLC1A5, TFRC, NCOA4, LPCAT3, GLS2, DPP4, CS, CARS1, ATP5MC3, ALOX1 and ATL1 (Fig. 12A). Additionally, m6A-related genes were significantly upregulated in LIHC with high *VPS35* expression, including METTL3, METTL14, WTAP, VIRMA, RBM15, RBM15B, ZC3H13, METTL16, CBLL1, YTHDC1, YTHDC2, YTHDF3, YTHDF1, YTHDF2, HNRDF2, HNRNPC, IGF2BP1, IGF2BP2, IGF2BP3, RBMX, EIF3A, HNRNPA2B1, FTO, ALKBH5 and ALKBH3 (Fig. 12B).

### Molecular docking of VPS35

**Fig. 13:**
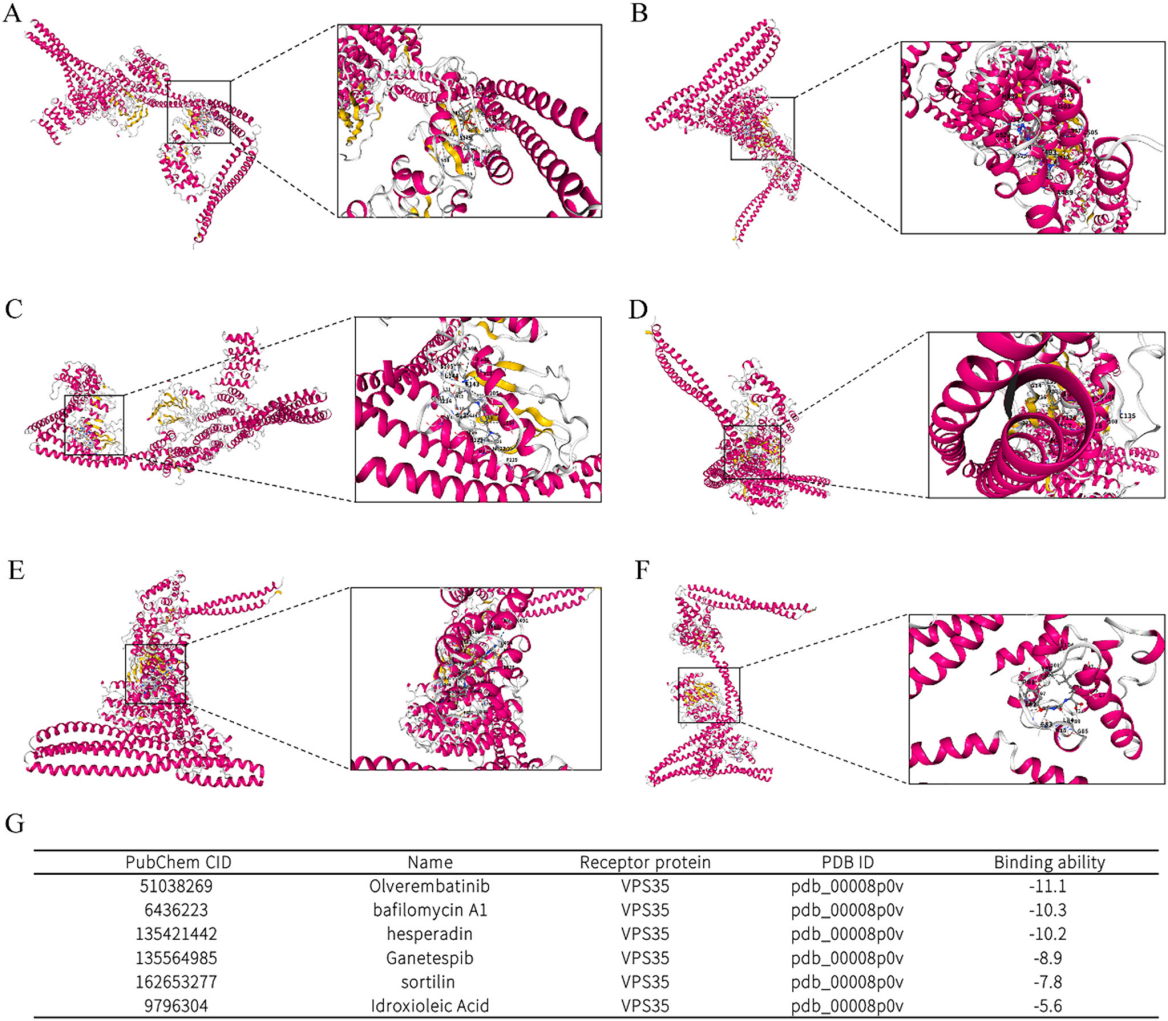
Molecular docking of VPS35 with potential ligands. (A) The binding sites of VPS35 and olverembatinib. (B) Bafilomycin A1. (C) Hesperadin. (D) Ganetespib. (E) Sortilin. (F) Idroxiolenic acid. (G) The binding ability of VPS35 to these compounds.

The molecular docking results showed that *VPS35* can bind to various compounds, with *VPS35* having the highest binding potential to six compounds: olverembatinib, bafilomycin A1, hesperadin, ganetespib, sortilin, and idroxiolenic acid (Fig. 13A–G).

### *In vitro* and *in vivo* validation of the biological function of VPS35 in LIHC

**Fig. 14:**
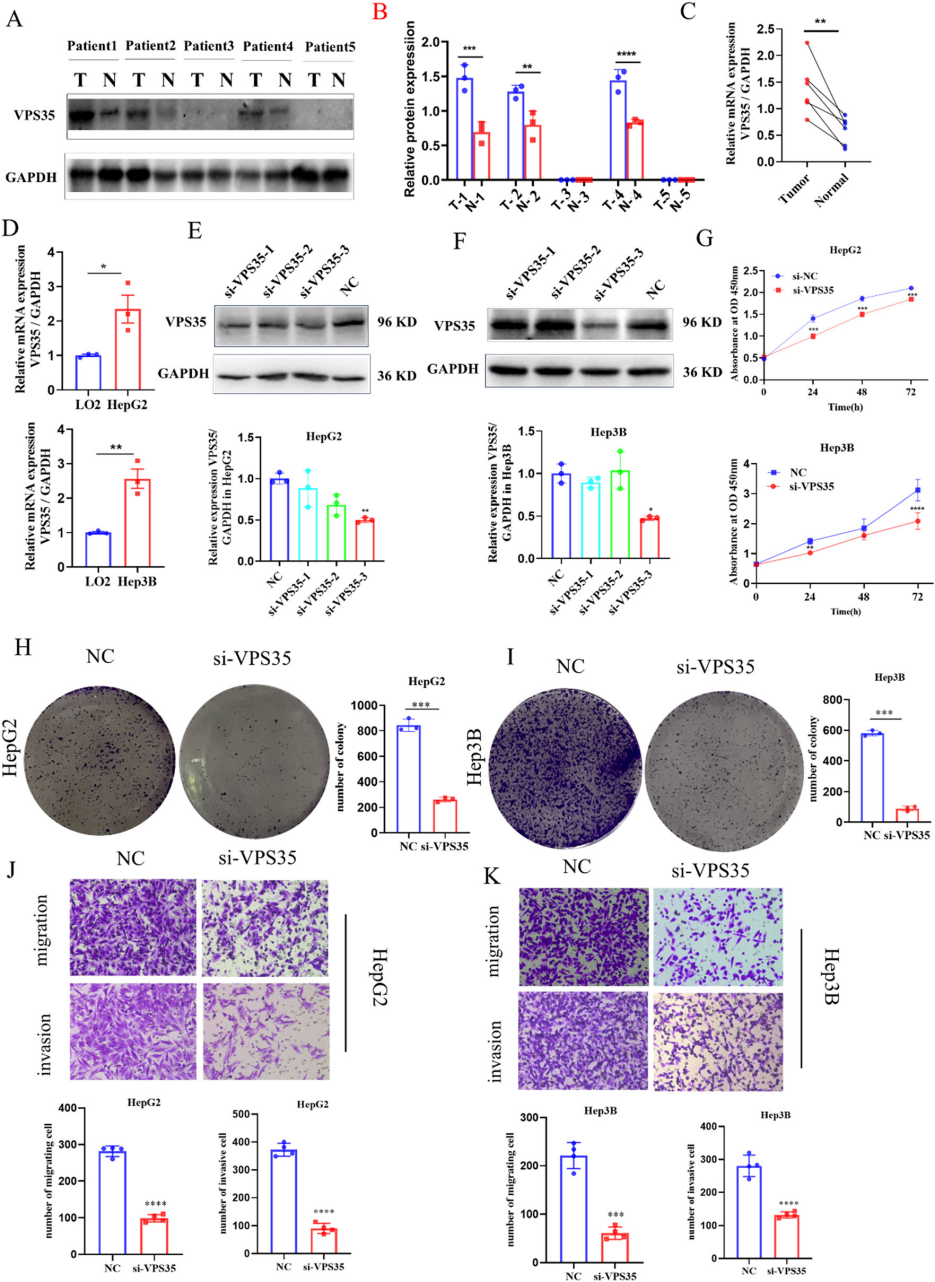
Functional validation of VPS35 in HCC *in vitro*. (A) Western blot detection of VPS35 protein expression levels in specimens from 5 pairs of HCC clinical specimens. (T: tumor, N: normal). (B) Quantitative analysis of VPS35 protein expression from A, band intensities were quantified by densitometry and normalized to GAPDH. (C) RT-qPCR detection of VPS35 mRNA expression levels in specimens from 5 pairs of HCC patients. (D) RT-qPCR detection of VPS35 mRNA expression levels in HepG2 and Hep3B cells. (E and F) Western blot assay of HepG2 and Hep3B VPS35 knockdown cells. (G) CCK-8 assay was performed to determine the relationship between VPS35 expression and growth ability. (H and I) Colony formation assay was performed to determine the relationship between VPS35 expression and clone formation ability. (J and K) Migration and invasion assays were conducted to determine the relationship between VPS35 expression and cell migration and invasion ability (ns: no significance; * *p* < 0.05; ** *p* < 0.01; *** *p* < 0.001). The data of all the above experiments are presented as the average values ± standard deviations of three independent experiments (*n* = 3).

Based on bioanalytical studies, we conducted a series of validation experiments to confirm the reliability of the results. We performed WB and RT-qPCR assays on the collected LIHC tissues and paired normal tissues, and the results demonstrated that VPS35 was significant highly expressed in LIHC (Fig. 14A–C). Then, at the cellular level, we validated the expression of VPS35 through RT-qPCR assays (Fig. 14D). In order to carry out subsequent cell biology experiments, we performed a siRNA-mediated loss-of-function approach to better understand the role of *VPS35* in LIHC biology. To identify the most effective siRNA, we transfected HepG-2 and Hep3B cells with each of the three *si-VPS35* constructs separately. Knockdown efficiency was evaluated at the protein level by Western blot analysis (Fig. 14E and F). *si-VPS35-3* demonstrated the highest knockdown efficiency in both cell lines. Therefore, *si-VPS35-3* was selected for all subsequent functional experiments. Subsequently, a CCK-8 assay was conducted to evaluate cell proliferation, demonstrating that decreased *VPS35* expression hindered cell proliferation (Fig. 14G). Furthermore, a colony formation assay indicated that reduced *VPS35* expression impaired the cells' ability to form colonies (Fig. 14H and I). Consistently, cell migration and invasion capabilities were notably inhibited in VPS35-silenced HepG2 and Hep3B cells (Fig. 14J and K). In conclusion, these findings indicate that *VPS35* plays a critical function in regulating the proliferation, migration, and invasion capacities of HepG2 and Hep3B cells *in vitro*.

**Fig. 15:**
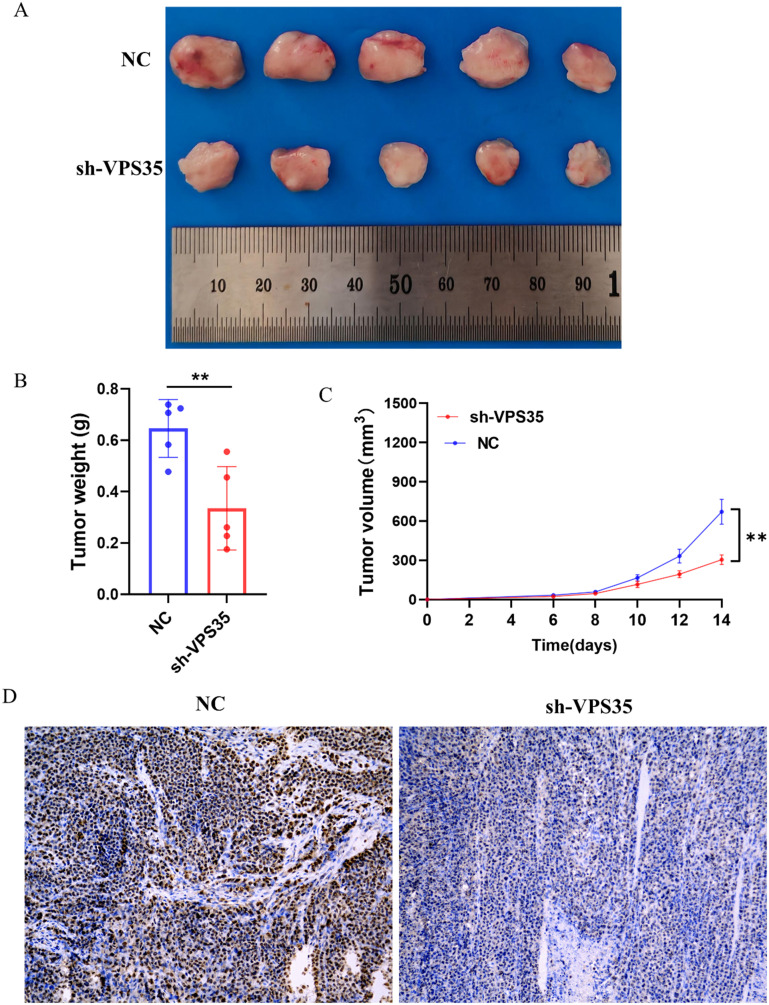
VPS35 promotes the proliferation of HepG2 cells *in vivo*. (A) In subcutaneous tumorigenesis experiments with nude mice, the size of the tumor was detected after VPS35 knockdown. (B) The weight of the tumor was detected after VPS35 knockdown. (C) The volume of the tumor was detected after VPS35 knockdown in 14 days. (D) Immunohistochemical experiments were used to detect the proliferation of HepG2 cells *in vivo* after VPS35 knockdown (ns: no significance; * *p* < 0.05; ** *p* < 0.01; *** *p* < 0.001).

6-Weeks-old nude mice were experimentally studied with subcutaneous xenografts. Since the HepG2 cells were injected under the skin of the mice, we measured the volume of the tumors every other day, after 14 days we dissected out the tumors from the mice. The result is as shown in Fig. 15A–C; the size, volume, and weight of the tumor all decreased after knockdown of *VPS35*. Next, we performed immunohistochemical staining of KI67 on the tumor tissue and found that cell proliferation decreased after the knockdown of *VPS35* (Fig. 15D).

### Discussion

VPS35 is a retromer protein primarily localized in endosomes and lysosomes,^28^ and it has been discovered that *VPS35*, as an essential structural component of the retromer complex, can regulate tumor growth and metastasis in liver and breast cancer by modulating multiple signaling pathways.^25^ However, the relationship between *VPS35* and LIHC has not been comprehensively reported. In this study, combining biological information analysis with *in vitro* cell assays and *in vivo* experiments, we aim to investigate the potential association between *VPS35* and tumor immunity, as well as LIHC promotion.

Biomarkers play a pivotal role in tumor prognosis assessment. To evaluate the diagnostic potential of *VPS35* in LIHC, we performed a ROC (receiver operating characteristic) curve analysis, which yielded an AUC (area under the curve) of 0.868, indicating strong diagnostic performance. Furthermore, using TCGA-LIHC (liver hepatocellular carcinoma) data, we conducted a Cox regression analysis, which identified *VPS35* as an independent risk factor for LIHC development. Additionally, Kaplan–Meier survival analysis revealed that high *VPS35* expression was significantly correlated with shorter overall survival (OS) in LIHC patients, further supporting its association with poor prognosis. In conclusion, our findings collectively suggest that *VPS35* holds significant diagnostic and prognostic value in LIHC.

Recent evidence demonstrates that MSI is a predictive biomarker for immunotherapy.^29–32^ In addition to MSI, tumor mutational burden (TMB) has been investigated as a potential biomarker for predicting response to immunotherapy.^33,34^ Clinical evidence shows a positive association between elevated TMB/MSI levels in tumor tissue and enhanced immunotherapy efficacy.^35,36^ Aligning with these observations, *VPS35* expression demonstrated significant positive associations with both TMB and MSI levels across LIHC. These data suggest that elevated *VPS35* expression might predict superior post-immunotherapy survival outcomes in LIHC. This discovery underscores *VPS35*'s promise as an emerging immunomodulatory target in oncology.

Mounting evidence indicates that the infiltrating immune cells within the tumor immune microenvironment (TIME) play crucial roles in tumor development and progression, thereby affecting the prognosis of tumor patients.^37–39^ In this study, we reported that *VPS35* expression was significantly correlated with the infiltration of effector memory T cells (Tem), CD4 cells, Th2 cells, and immature dendritic (iDC) cells. Moreover, *VPS35* expression was positively associated with chemokines CCL25, CCL14, and CCL21, and receptors CCR7, CXCR1, and CXCR3 in LIHC. Furthermore, we found that *VPS35* is involved in multiple immune-related processes, suggesting that *VPS35* may play a key regulatory role in immune-related pathways. Together, these findings suggest that *VPS35* may serve as a key regulator in tumor immunity and a potential biomarker associated with immune infiltration in LIHC. However, the mechanisms by which *VPS35* influences immune cell infiltration remain incompletely elucidated, necessitating further in-depth investigations to clarify its exact role in the TIME.

In recent years, immune checkpoint inhibitor (ICI) therapy has emerged as a standard of care in the cancer treatment landscape beyond radiotherapy and chemotherapy.^40,41^ However, higher TIDE scores correlate with increased tumor immune evasion, leading to reduced response rates to ICI therapy.^42,43^ Therefore, we employed the TIDE score to predict ICI-related markers such as PD-L1/CD274. The significantly lower TIDE score for PD-L1 in the *VPS35*-low group implies that tumors with low *VPS35* may be more susceptible to immune checkpoint blockade, possibly due to a less exhausted or more infiltrated T-cell state. This nominates *VPS35* as a novel, potential predictive biomarker for immunotherapy in LIHC, meriting further clinical investigation.

Our analysis of chemotherapy response revealed a nuanced relationship between *VPS35* expression and drug sensitivity. While the *VPS35*-high group exhibited greater sensitivity to certain agents such as axitinib, it concurrently demonstrated higher resistance to others, including cisplatin, as indicated by elevated IC_50_ values. This apparent contradiction underscores the complexity of VPS35's functional roles in tumor biology and cautions against an oversimplified interpretation of its therapeutic potential. Therefore, while *VPS35* represents a promising biomarker for predicting treatment response, its value lies in stratifying patients for specific therapies rather than being a unilateral indicator of general chemosensitivity. Further research using isogenic cell models with modulated *VPS35* expression is warranted to dissect the precise mechanisms underlying these distinct drug sensitivity profiles.

Ferroptosis is a novel form of regulated cell death characterized by iron-dependent accumulation of lipid peroxides, distinct from apoptosis, necrosis, and autophagy. Ferroptosis plays a pivotal role in the pathogenesis of hepatocellular carcinoma.^44^ Relevant studies suggested that the abnormal expression of m6A-related proteins is related to tumor occurrence and development.^45^ The unregulated expression of m6A-related genes may indicate the poor prognosis of LIHC. In this study, the results demonstrate that ferroptosis-related and m6A-related genes were significantly upregulated in LIHC with high *VPS35* expression. These findings suggest that the strong positive correlations between *VPS35* and key regulators of ferroptosis and RNA m6A modification open new avenues for research, suggesting that *VPS35* may also influence LIHC progression through regulating these emerging hallmarks of cancer.

We confirmed the significant upregulation of *VPS35* in LIHC at the mRNA, protein, and spatial transcriptome levels, a finding consistent across multiple independent databases and our own clinical samples. This overexpression was not an epiphenomenon but was closely linked to aggressive clinicopathological features and served as an independent predictor of poor survival. To confirm these findings, siRNA was transfected into two liver cancer cell lines to knock down *VPS35*. The results showed that the knockdown of *VPS35* inhibited cell proliferation, migration, and invasion. Furthermore, we also conducted experiments in animal bodies, and the results showed that the size, volume and weight of the tumor all decreased after the knockdown of *VPS35*.

Although our study primarily focused on establishing the clinical and prognostic relevance of *VPS35* in LIHC, the underlying molecular mechanisms warrant further exploration. Based on our bioinformatics analyses and supporting literature, we propose several plausible pathways through which *VPS35* may exert its oncogenic functions. Our GSEA results indicated that high *VPS35* expression was significantly associated with processes such as “homophilic cell adhesion *via* plasma membrane adhesion molecules” and “immunoglobulin receptor binding,” suggesting a potential role for *VPS35* in recycling adhesion receptors (*e.g.*, E-cadherin, integrins) or immune-related receptors, thereby influencing cell–cell interactions, metastasis, and immune modulation within the tumor microenvironment. Furthermore, our PPI and co-expression network analyses identified several key interactors and co-expressed genes, including filamin A (FLNA)^46^ and pyruvate dehydrogenase kinase 1 (PDK1),^47^ which are well-established regulators of cytoskeletal dynamics and the PI3K/AKT signaling pathway, respectively. The strong correlation between *VPS35* and these signaling molecules implies a potential mechanism whereby *VPS35* facilitates tumor progression by regulating critical signaling hubs. This notion is further supported by previous studies in other cancer types: for instance, *VPS35* has been shown to promote gastric cancer proliferation by recycling EGFR to the cell surface, leading to sustained activation of the ERK1/2 pathway.^14^ Similarly, in LIHC, *VPS35* has been linked to the Wnt/PCP^14^ and β-catenin pathways.^17^ Thus, while the exact cargoes of *VPS35* in LIHC remain to be fully elucidated, our data strongly suggest that *VPS35* may promote tumor progression by modulating the trafficking of key receptors and signaling molecules, thereby activating downstream pathways such as PI3K/AKT, ERK, and Wnt/β-catenin. Further research should aim to experimentally validate these potential cargoes and their functional impact on LIHC pathogenesis.

However, our study still has several limitations. Firstly, the clinical cohort size remains limited for robust subgroup analysis. Secondly, while we have established a clear correlation between *VPS35* and TIME, chemotherapy response, ferroptosis, and m6A, the precise molecular mechanisms underlying these relationships remain largely speculative. Thirdly, while our computational analyses using reputable algorithms strongly suggest that *VPS35* expression may influence response to immunotherapy and chemotherapy, these findings require rigorous validation in future studies. This includes retrospective analysis of patients with LIHC treated with ICIs or specific chemotherapeutic agents to test the combination of *VPS35* targeting with standard-of-care treatments. So further studies should be conducted to investigate the specific role and potential molecular mechanisms of *VPS35* in tumorigenesis, progression, and immune infiltration. The inability to obtain patients' detailed history of co-morbidities (*e.g.*, hepatitis viral loads, hepatic function indexes), which may affect the expression of *VPS35*. Future research will require the inclusion of larger cohorts and additional evidence for in-depth investigation to validate and expand upon these findings, thereby enhancing reliability for clinical applications.

## Author contributions

S. Z. and X. L. selected the research topic and conducted the design of this topic. F. L. and Q. W. carried out the collection of clinical samples. H. Z. carried out the statistical analysis. D. W. and Y. Q. conducted experiments and bioinformatics analysis and wrote the first draft of this manuscript. All authors have read and agreed to the published version of the manuscript.

## Conflicts of interest

The authors have declared that no competing interest exists.

## Supplementary Material

MD-OLF-D5MD00834D-s001

## Data Availability

All data generated or analysed during this study are included in this published article and its supplementary information (SI) files. Supplementary information: the expression of *VPS35* in human organs and tissues,Kaplan-Meier curve analysis of the OS of *VPS35* in different clinical subtypes and the immunotherapy and chemotherapy response analysis of *VPS35*. See DOI: https://doi.org/10.1039/d5md00834d.
