# Supplementary material for: Comprehensive multi-omics analysis and experimental validation indicate that VPS35 is a promising biomarker for prognosis, immunotherapy, and chemotherapy in LIHC
Source: RSC Med Chem. 2026 Jan 12;17(2):1107–34. doi: 10.1039/d5md00834d (PMC12794317; doi:10.1039/d5md00834d)
Supplement: MD-017-D5MD00834D-s001 [file MD-017-D5MD00834D-s001.pdf]

Supplementary Information (SI) for RSC Medicinal Chemistry.
This journal is © The Royal Society of Chemistry 2025

Table S1. Details of LIHC patients

	Age	Gender	Clinical stages	Pathological grade	AFP(ng/ml)
Patient 1	65	Man	III	G2	110
Patient 2	82	Woman	III	G3	1430
Patient 3	45	Man	I	G1	480
Patient 4	33	Woman	II	G3	314
Patient 5	58	Man	I	G1	220.5

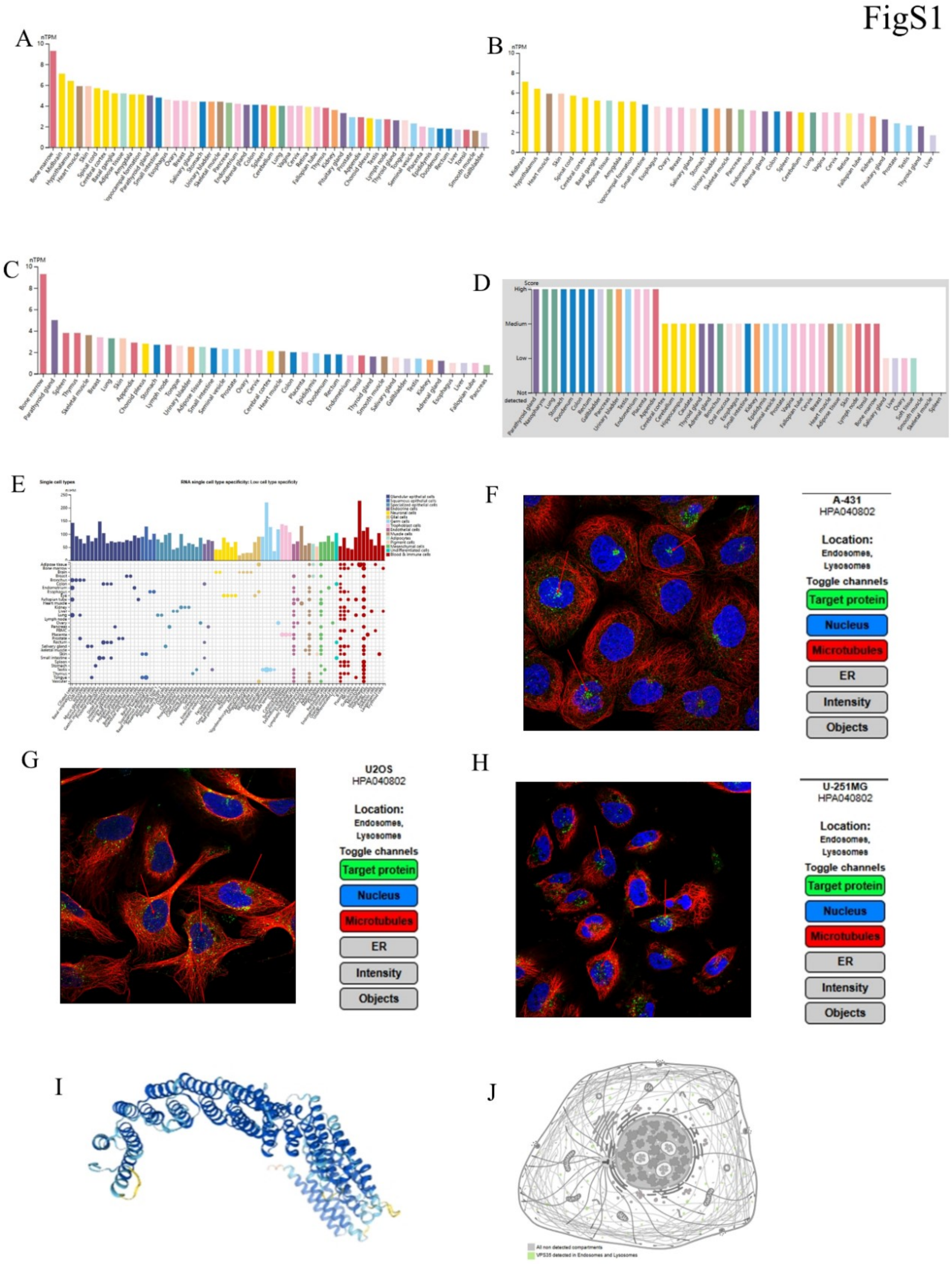

FigureS1. The expression of VPS35 in human organs and tissues. (A-C) Summarized VPS35 mRNA expression in various organs and tissues, based on the consensus, GTEx and HPA dataset. (D) VPS35 protein expression in human organs and tissues. (E) The expression of VPS35 in various single-cell tissues. (F-J) The subcellular localization of VPS35, as depicted by immunofluorescence visualization in HPA database.

FigS2

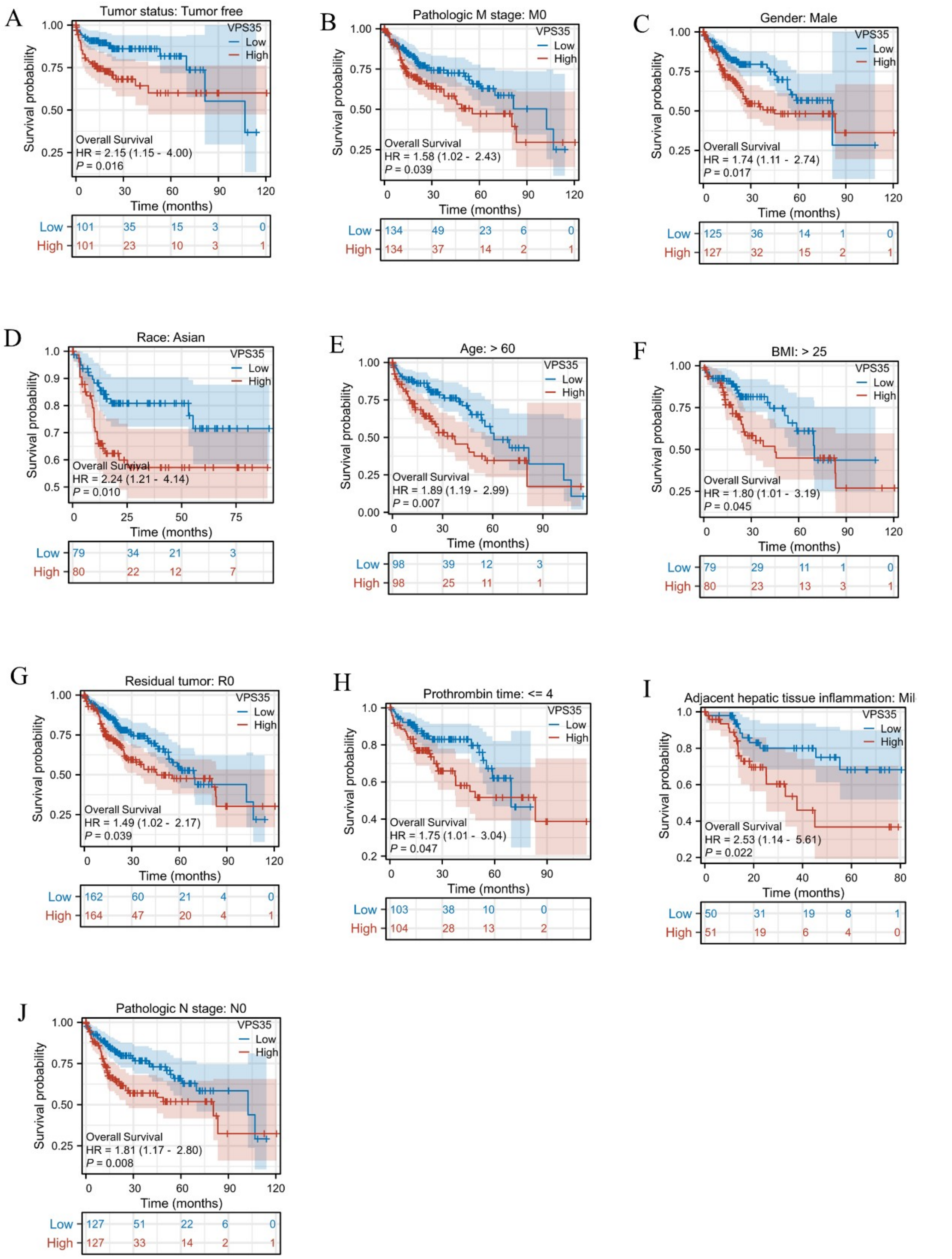

FigureS2. Kaplan-Meier curve analysis of the OS of VPS35 in different clinical subtypes. (A)Tumor status: Tumor free. (B)Pathologic M stage: M0. (C) Gender: male. (D) Race: Asian. (E) Age > 60. (F) BMI >25. (G) Residual tumor: Ro. (H)Prothrombin time <=4. (I)Adjacent hepatic tissue inflammation: Mil. (J)Pathologic N stage: N0.

FigS3

A

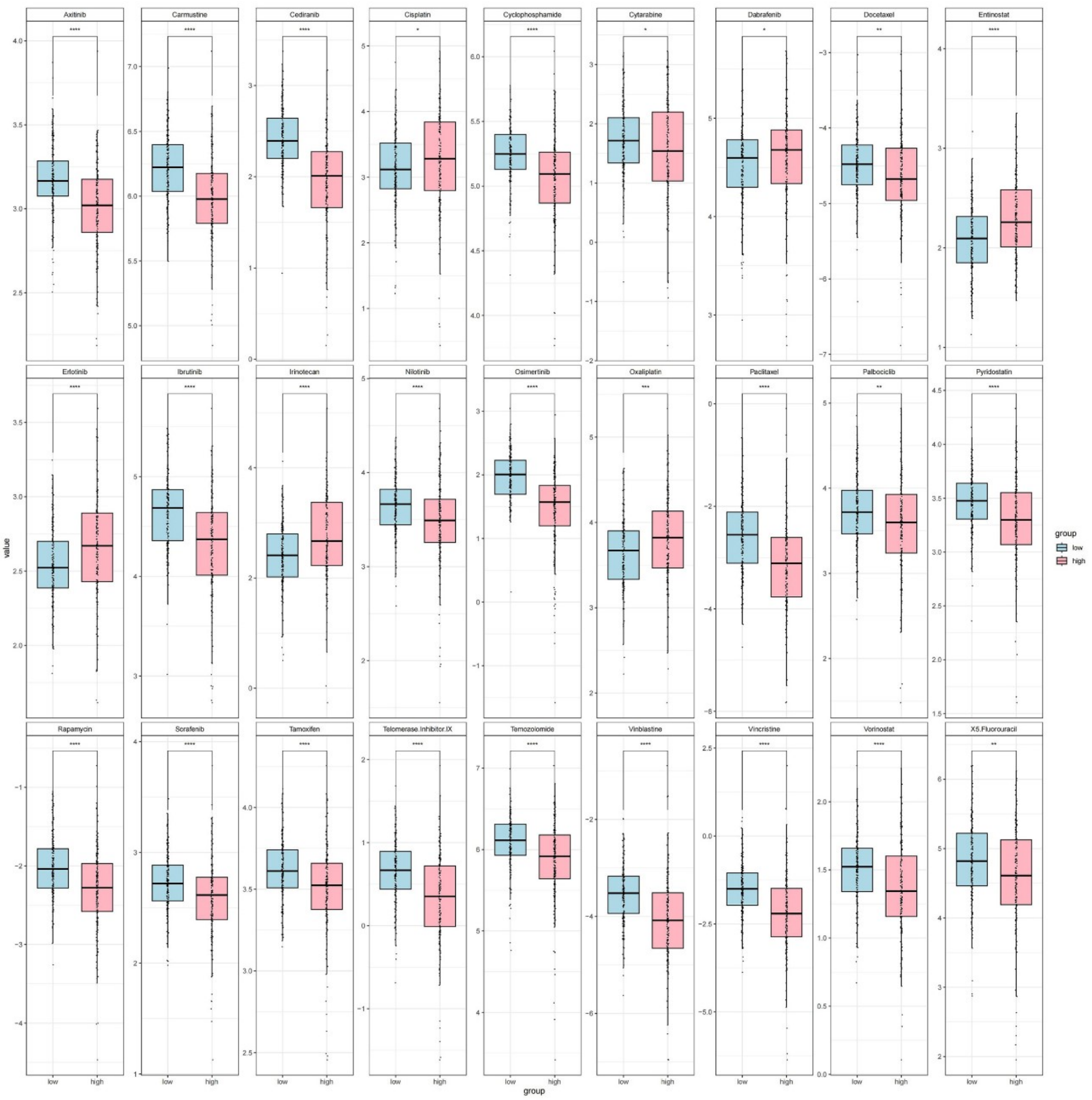

FigureS3. The immunotherapy and chemotherapy response analysis of VPS35. (A) The chemotherapeutic drug half-inhibitory concentration (IC50) values in VPS35-high and VPS35-low samples. (ns: no significance; * p < 0.05; ** p < 0.01; *** p < 0.001).
